# Sleep duration and napping in relation to colorectal and gastric cancer in the MCC-Spain study

**DOI:** 10.1038/s41598-021-91275-3

**Published:** 2021-06-03

**Authors:** Kyriaki Papantoniou, Gemma Castaño-Vinyals, Ana Espinosa, Michelle C. Turner, Vicente Martín-Sánchez, Delphine Casabonne, Nuria Aragonés, Inés Gómez-Acebo, Eva Ardanaz, Jose-Juan Jimenez-Moleon, Pilar Amiano, Ana Molina-Barceló, Juan Alguacil, Guillermo Fernández-Tardón, José María Huerta, Natalia Hernández-Segura, Beatriz Perez-Gomez, Javier Llorca, Juana Vidán-Alli, Rocıo Olmedo-Requena, Leire Gil, Carmen Castañon-López, Marina Pollan, Manolis Kogevinas, Victor Moreno

**Affiliations:** 1 Department of Epidemiology, Center of Public Health, Medical University of Vienna, Kinderspitalgasse 15, 1090 Vienna, Austria; 2 ISGlobal, Barcelona, Spain; 3 Universitat Pompeu Fabra (UPF), Barcelona, Spain; 4 Consortium for Biomedical Research in Epidemiology and Public Health (CIBERESP), Madrid, Spain; 5 IMIM (Hospital del Mar Medical Research Institute), Barcelona, Spain; 6 McLaughlin Centre for Population Health Risk Assessment, University of Ottawa, Ottawa, ON Canada; 7 Biomedicine Institute (IBIOMED), University of León, Leon, Spain; 8 Unit of Molecular and Genetic Epidemiology in Infections and Cancer (UNIC-Molecular), Cancer Epidemiology Research Programme, IDIBELL, Catalan Institute of Oncology, Hospitalet de Llobregat, Barcelona, Spain; 9 Epidemiology Section, Public Health Division, Department of Health of Madrid, Madrid, Spain; 10 University of Cantabria, Santander, Spain; 11 IDIVAL, Santander, Spain; 12 Navarra Public Health Institute, Pamplona, Spain; 13 IdiSNA, Navarra Institute for Health Research, Pamplona, Spain; 14 Department of Preventive Medicine and Public Health, University of Granada, Granada, Spain; 15 Instituto de Investigacion Biosanitaria de Granada (Ibs.GRANADA), Hospitales Universitarios de Granada/Universidad de Granada, Granada, Spain; 16 Public Health Division of Gipuzkoa, San Sebastian, Spain; 17 Biodonostia Research Institute, San Sebastian, Spain; 18 Cancer and Public Health Area, FISABIO-Public Health, Valencia, Spain; 19 Centro de Investigación en Salud y Medio Ambiente (CYSMA), Universidad de Huelva, Campus Universitario de El Carmen, Huelva, Spain; 20 Health Research Institute of the Principality of Asturias, University of Oviedo, Oviedo, Spain; 21 Department of Epidemiology, Murcia Regional Health Council, IMIB-Arrixaca, Murcia, Spain; 22 Cancer Epidemiology Unit of the National Center for Epidemiology, Carlos III Institute of Health, Madrid, Spain; 23 Cancer Epidemiology Research Group, Oncology and Hematology Area, IIS Puerta de Hierro (IDIPHIM), Majadahonda, Madrid, Spain; 24 Servicio de Oncología, Complejo Asistencial Universitario de León, Leon, Spain; 25 Unit of Biomarkers and Susceptibility, Oncology Data Analytics Program, Catalan Institute of Oncology (ICO), Hospitalet de Llobregat, Barcelona, Spain; 26 Colorectal Cancer Group, ONCOBELL Program, Bellvitge Biomedical Research Institute (IDIBELL), Hospitalet de Llobregat, Barcelona, Spain; 27 Department of Clinical Sciences, Faculty of Medicine, University of Barcelona, Barcelona, Spain

**Keywords:** Cancer, Gastroenterology, Risk factors

## Abstract

Sleep duration is a novel and potentially modifiable risk factor for cancer. We evaluated the association of self-reported sleep duration and daytime napping with odds of colorectal and gastric cancer. We included 2008 incident colorectal cancer cases, 542 gastric cancer cases and 3622 frequency-matched population controls, recruited in the MCC-Spain case–control study (2008–2013). Sleep information, socio-demographic and lifestyle characteristics were obtained through personal interviews. Multivariable adjusted logistic regression models were used to estimate odds ratios (OR) with 95% confidence intervals (CI) for cancer, across categories of sleep duration (≤ 5, 6, 7, 8, ≥ 9 hours/day), daytime napping frequency (naps/week) and duration (minutes/nap). Compared to 7 hours of sleep, long sleep was associated with increased odds of colorectal (OR_≥9 hours_: 1.59; 95%CI 1.30–1.94) and gastric cancer (OR_≥9 hours_: 1.95; 1.37–2.76); short sleep was associated with increased odds of gastric cancer (OR_≤5 hours_: 1.32; 0.93–1.88). Frequent and long daytime naps increased the odds of colorectal (OR_6–7 naps/week, ≥30 min_: 1.32; 1.14–1.54) and gastric cancer (OR_6–7 naps/week, ≥30 min_: 1.56; 1.21–2.02). Effects of short sleep and frequent long naps were stronger among participants with night shift-work history. Sleep and circadian disruption may jointly play a role in the etiology of colorectal and gastric cancer.

## Introduction

Colorectal cancer (CRC) is the third most common cancer worldwide (1,800,977 new cases in 2018), and the second most common cause of cancer death (861,663 deaths in 2018)^1^. Gastric cancer is the sixth most commonly diagnosed cancer (1,033,701 new cases) and third cause of cancer death (782,685 deaths)^1^. CRC incidence rates overall have declined since the mid-1980s because of changing patterns in risk factors and screening^2^. However, CRC occurrence has been on the rise in patients younger than age 50 for unknown reasons^3^. Despite extensive research on the etiology and advancements in early detection and treatment of colorectal and gastric tumors, more research on identifying modifiable risk factors is needed^2^.

Sleep duration has been suggested as a novel and potentially modifiable risk factor for chronic disease incidence and mortality^4–6^. Specifically, short and long sleep duration have been associated with a higher risk for cardiovascular disease^7^, type II diabetes^8,9^, obesity^9,10^ and cancer^11–13^. Most studies of cancer so far have focused on the effects of sleep duration on breast cancer and the evidence on other tumors is limited^14,15^. Long sleep duration has been associated with an increased risk of colorectal cancer in two prospective cohorts of health professionals, especially among individuals who were overweight or snored regularly^16^. Long and short sleep were both associated with an elevated risk of CRC in a cohort study of postmenopausal women^17^. So far only one study, a prospective cohort of older adults, has reported an increased risk for gastric cancer associated with short sleep^18^. In a recent study prediagnosis short sleep and long napping were associated with significantly higher mortality among CRC survivors^19^. No previous study has, to our knowledge, evaluated daytime napping as a risk factor for colorectal and gastric cancer.

Our aim in the present study was to evaluate habitual sleep duration and daytime napping in relation to colorectal and gastric cancer adjusting for potential confounders and other sleep characteristics in a large population based case–control study. Long-term night shift work has been shown to increase the odds of colorectal and gastric cancer in our previous analyses^20,21^. Therefore, we additionally conducted stratified analyses by night shift work history to evaluate potential joint effects of sleep and circadian disruption in relation to gastrointestinal cancer risk.

## Methods

The MCC-Spain Study is a population-based case–control study on five tumors (breast, colorectal, prostate, stomach and chronic lymphocytic leukemia) in Spain, with the general aim to investigate environmental and genetic factors related to cancer risk. Methods used in this study have been previously reported in detail^22^. In brief, the study recruited incident cancer cases from 23 hospitals in 12 different regions in Spain and a common set of population controls for all cases. The recruitment of cancer cases and controls study began in the year 2008 and was conducted through 2013. All procedures performed in studies involving human participants were in accordance with the ethical standards of the research committee, and with the 1964 Helsinki Declaration and its later amendments or comparable ethical standards. The protocol of MCC-Spain was approved by each of the ethics committees of the participating institutions (Ethical Committee of Clinical Research of Barcelona, Cantabria, Girona, Gipuzkoa, Huelva, León, Principado de Asturias, Madrid, Navarra and Valencia). All participants provided informed consent prior to enrolment into the study. The data underlying this article cannot be shared publicly for the privacy of individuals that participated in the study. The data can be shared on reasonable request to the corresponding author and coordinator of the study.

### Study population

Cases were males and females, aged 20–85 years with a new histological confirmed diagnosis of colorectal or gastric cancer living in the catchment area of each hospital for at least 6 months. Cases were defined by the International Classification of Disease 10 (ICD-10) codes: C18, C19, C20, D01.0, D01.1, D01.2 for colon or rectum, C16 and D00.2 for stomach and C15.5 for cancer cases of the lower third of the oesophagus. Clinical data such as CRC histological type (adenocarcinoma, mucinous adenocarcinoma, signet ring-cell carcinoma, squamous cell carcinoma, medullary carcinoma, undifferentiated carcinoma, other, type not specified), tumor localization (proximal colon, distal colon, rectum), and TNM staging information was available through hospital medical records. Most CRC tumors were adenocarcinomas (92% adenocarcinomas, 5.5% mucinous carcinomas, 1.5% other types, 1% not specified). Most colorectal cancers (52%) were at clinical stage II or lower at recruitment, and in 110 patients (5.3%) we could not establish the clinical stage. Clinical data for gastric cancer included tumor histological type, localization, Lauren classification, the 2010 classification of the WHO and degree of differentiation Data on *Helicobacter pylori* (*H. pylori*) status were available in 279 cases, with 93% positive. Controls living in the same catchment area as cases for the same period of time (≥ 6 months) were randomly selected from the rosters of General Practitioners (GP) at the Primary Health Centers (PHC) included in the study. They were contacted on behalf of their GP and invited to participate in the study. Controls were frequency-matched to the overall distribution of cases, by age, sex and center, ensuring that in each center there was at least one control of the same sex and 5-year interval for each case. Eligible controls were free of CRC or gastric cancer history, respectively. Subjects were ineligible if they were incapable to participate in the interview due to communication difficulties (e.g. speaking problems) or excess impairment of physical ability. Response rates among cases and controls varied by center and on average were 68% among colorectal cancer cases, 55% among gastric cancer cases and 54% among controls with valid telephone numbers. There were no differences in educational level and age between those subjects who participated and those who did not respond to the study invitation or refused to participate. A total of 2140 CRC cases, 459 gastric cancer cases, 3950 controls for colorectal cancer and 3440 controls for gastric cancer were recruited in the study. Participants with missing information on sleep duration (N = 487, 7.3%) and key confounders (N = 7, 0.1%) were excluded. The present analysis included 2008 colorectal cancer cases, 452 gastric cancer cases, 3598 controls for colorectal cancer and 3099 population controls for gastric cancer (3077 were common controls used in both analyses).

### Data collection

Participant information was collected using face-to-face interviews performed by trained personnel. Interviews with cancer patients were scheduled shortly after a new cancer diagnosis. The median time between date of diagnosis and interview was 63 days (interquartile range, iqr 106) for colorectal cancer and 40 days (iqr 75) for gastric cancer. To evaluate sleep duration, subjects were asked to report how many hours on average they slept per night at the time of recruitment. They were also asked how frequently (days/week) they usually took daytime naps (“siestas”) and what was their average duration (minutes/nap). Sleep quality was assessed using questions on occurrence of sleep problems (difficulty falling asleep, waking up during the night, taking sleep medication) that lasted for at least 1 year through lifetime and also by reporting the the age of beginning and end of the sleep problem. Sleep timing was assessed asking participants to report their habitual bedtime, if they reported going to sleep at approximately the same time every day during the last 10 years, and whether they had experienced a period of at least 1 year with frequent changes in their bedtime. Night shift work (working for at least 3 h between midnight and 5am at least 3 times per month) was assessed using a lifetime occupational history and detailed questions on work schedules for all jobs held for at least a year. Information on known or suspected risk factors for colorectal and gastric cancer was collected including age, educational level, family socioeconomic level, race, BMI, family history of colorectal and gastric cancer, smoking status, physical activity, medication use, and personal disease history (e.g. diabetes). In a subset of participants (88% of controls, 88% of colorectal cancer cases, 77% of gastric cancer cases) a self-administered diet questionnaire was used to assess current and past (at the age of 30–40 years) diet habits including alcohol consumption.

### Statistical analysis

In bivariate analyses we examined participant’s characteristics by case–control status and by categories of sleep duration and daytime napping. We used generalized additive models (GAM) to inspect the linearity of associations of sleep duration with CRC and gastric cancer. GAMs used a smoothing function for sleep duration and were adjusted for confounders. We used unconditional logistic regression analysis and calculated odds ratios (OR) with 95% confidence intervals (CI) for colorectal and gastric cancer separately, across predefined categories of : (i) nighttime sleep duration (≤ 5 hours, 6 hours, 7 hours (reference), 8 hours, ≥ 9 hours), (ii) daytime napping frequency (no napping (reference), 1–5 days/week, 6–7 days/week), (iii) daytime napping duration (no napping, < 15 min, 15–29 min, 30–59 min, ≥ 60 min), (iv) combined napping frequency and duration (no napping, 1–5 naps/week for < 30 min, 6–7 naps/week for < 30 min, 1–5 naps/week for ≥ 30 min, 6–7 naps/week for ≥ 30 min). For the combined analysis, the median of napping duration (30 min) and the 25th percentile of napping frequency (5 days/week) among controls that napped were used as cut-offs. We also assessed the effects of ever having sleep problems (for at least 1 year), duration of sleep problems (years) and having experienced frequent changes in bedtime. Crude models were adjusted for frequency-matched variables (age, center, sex) and educational level (less than primary, primary, high school, university). Fully-adjusted models were additionally adjusted for family history of CRC or gastric cancer in first degree relatives (yes/no), body mass index (< 25, 25–29, ≥ 30 units), leisure time physical activity (inactive, little active, moderately active, very active)), smoking status (never, ex-smoker, current smoker) and occupational status (employed, unemployed, housewives, retired). A category of missing values was used for confounders included in the final model. Full case-analysis was performed in a subset of participants (88% of controls, 88% of colorectal cancer cases, 77% of gastric cancer cases), with diet information on usual and past dietary intake. In this analysis we additionally adjusted for diet habits [past alcohol consumption (quartiles), total energy intake in grams/day (quartiles), red meat consumption in grams/day (quartiles), fruit consumption in grams/day (quartiles), vegetable consumption in grams/day (quartiles)], other sleep characteristics (sleep duration/daytime napping; sleep problems, frequent changes in bedtime), and night shift work history (never shift work, permanent night shifts, rotating night shifts, rotating shifts, housewives). Further adjustment to the above mentioned variables made minimal difference in risk estimates and these results are presented in Supplemental Table 1. We tested possible interactions between sleep duration/daytime napping and sex (women, men), BMI (< 25, 25–30, > 30), night shift work history (never, ever), education (primary school or less vs high school, university or higher) and age at recruitment/diagnosis (< 50 years, ≥ 50 years) using the likelihood ratio test (LRT) and performed stratified analyses. Stratified analysis by night shift work history (never, ever) was conducted among participants with night shift work information (87.5%), after excluding participants with missing night shift work history (CRC: 7.9%, gastric: 5.8%) and housewives (CRC/gastric: 6.7%). We also evaluated the association between sleep duration and daytime napping and anatomical subtypes (colon, rectum), and TNM staging (0–II, III, IV) of colorectal cancer and anatomical site (non-cardia/oesophageal, cardia) and Lauren classification (Intestinal vs Diffuse) for gastric cancer using multinomial logistic regression and tested for heterogeneity across subtypes of cancer. In sensitivity analysis we (i) excluded participants with any report of sleep problem in the 5 years prior to recruitment in the study (N = 1930, 31.7%) (ii) excluded retired workers (N = 3146, 52%), (iii) excluded participants with more than 6 months between the date of diagnosis and interview. Analyses were performed using Stata version 14.1.

**Table 1:** Characteristics of controls and cases with sleep duration information in the MCC-Spain study (N = 6082).

	Controls for colorectal cancer (N = 3598)^a^	Colorectal cancer cases (N = 2008)	Controls for gastric cancer (N = 3099)^a^	Gastric cancer cases (N = 452)
**Characteristics**				
Age; mean (SD)	63.2 (11.8)	66.9 (10.8)	64.0 (11.5)	66.4 (12.4)
BMI (kg/m^2^); mean (SD)	26.7 (4.5)	27.5 (4.5)	26.9 (4.4)	27.6 (4.2)
Women (%)	49.4	36.3	45.2	33.0
Postmenopausal (%)	72.7	90.0	75.1	88.6
Obese (%)	21.1	25.5	21.5	25.7
Primary school or less (%)	52.3	69.1	54.0	68.8
Never smokers (%)	44.5	41.3	44.2	40.7
Physically inactive (METS hour/week) (%)	40.7	48.9	42.0	50.7
Family history of colorectal cancer (%)	8.2	16.6	8.6	10.6
Family history of gastric cancer (%)	6.0	6.5	6.1	15.5
Night shift work history (%)	16.8	16.9	17.1	19.5
Retired (%)	48.0	58.1	51.6	53.5
**Diet habits; mean (SD)**^**b**^				
Total energy intake (kcal/day)	1896 (637)	2010 (704)	1907 (640)	2183 (858)
Past alcohol consumption (g ethanol /day)	17.3 (27.0)	24.3 (34.1)	18.1 (27.5)	27.8 (24.2)
All red meat consumption (g/day)	61.8 (38.8)	74.0 (48.7)	62.9 (38.8)	84.6 (54.8)
Fruit consumption (g/day)	349 (217)	345 (204)	353 (218)	356 (234)
Vegetable consumption (g/day)	190 (123)	172 (109)	192 (126)	181 (126)
**Sleep characteristics**				
Sleep duration; mean (SD)	7.0 (1.4)	7.3 (1.6)	7.0 (1.4)	7.2 (1.6)
Daytime napping (%)	54.6	61.2	56.6	59.7
Daytime napping frequency (days/week); mean (SD)^c^	5.8 (2.0)	6.1 (1.8)	5.9 (2.0)	6.1 (1.8)
Daytime napping duration (minutes/day); mean (SD)^c^	34.3 (31.6)	42.4 (36.4)	35.4 (32.0)	42.4 (35.1)
Ever sleep problems (%)	36.1	33.6	34.8	28.8
Ever sleep medication (%)	21.1	18.0	20.5	16.7
Frequent changes in bed time (%)	18.4	19.2	18.2	18.8

Some column totals do not add up due to missing data.

*BMI *body mass index, *METS *metabolic equivalents, *SD* standard deviation.

^a^3077 of the controls are common controls for colorectal and gastric cancer.

^b^Study subset (3193 colorectal cancer controls, 1773 colorectal cancer cases, 349 gastric cancer cases and 2732 gastric cancer controls).

^c^Computed among participants who reported daytime napping.

### Compliance with ethical standards

Ethical approval was obtained from the local ethical committee. Informed consent was obtained from all participants. All methods were carried out in accordance with relevant guidelines and regulations.

## Results

CRC cases were more likely to be older, obese, less physically active, to smoke, to have a lower educational level and to report CRC in their family history, than controls (Table 1). Similarly, gastric cancer cases were older and more likely to be obese, physically inactive, ever smokers, and to have family history of gastric cancer, compared to controls. Both colorectal and gastric cancer cases reported higher total energy intake, past alcohol consumption and red meat consumption compared to their respective controls. Average sleep duration was slightly longer among cases (CRC 7.3 h; SD1.6; gastric cancer 7.2 h; SD 1.6) than controls (7 h; SD 1.4). Daytime napping was reported among 61% of colorectal cancer cases, 60% of gastric cancer cases and approximately 55% of population controls. Both colorectal and gastric cancer cases were more likely to report a higher frequency (days/week) and duration (minutes/nap) of daytime napping compared to controls. Sleep problems and use of sleep medication was more frequent among controls than among cancer cases.

Controls in the extreme sleep duration categories (≤ 5 and ≥ 9 h) were more likely to be older and retired, have a higher BMI, lower education, and be less physically active, compared to those with 7 h of sleep (Table 2). Controls with the longest durations of sleep (≥ 9 h) were also more likely to be male, to have night shift work history and report higher past alcohol consumption, compared to participants with shorter sleep duration. Controls who slept ≤ 5 h were more likely to have sleep problems, take sleep medication, and report frequent changes in bed time. Daytime napping was less common in the extreme sleep duration categories, compared to the 7 h group, however frequency and duration of naps was higher in participants with extreme nighttime sleep duration, compared to 7 h of sleep. Participants napping 6–7 times a week were slightly older and more likely to be male, retired workers, smokers and less likely to have sleep problems. Sleep duration was similar between participants with and without napping and across categories of increasing napping frequency.

**Table 2:** Characteristics of sleep duration and daytime napping profiles among all controls included in the analysis of colorectal cancer (N = 3598).

Characteristics	Sleep duration (hours/day) (N = 3598)					Daytime napping frequency (N = 3496)		
	≤ 5	6	7	8	≥ 9	No naps	Napping 1–5 days/week	Napping 6–7 days/week
N	472	724	1048	987	368	1534	555	1407
Age; mean (SD)	65.1 (10.7)	62.5 (11.4)	60.4 (11.9)	63.7 (11.9)	68.8 (10.4)	62.1 (12.3)	58.4	66.4 (10.0)
BMI; mean (SD)	27.3 (4.7)	27.2 (4.6)	26.2 (4.2)	26.5 (4.3)	27.7 (4.8)	26.6 (4.6)	26.1 (4.2)	27.2 (4.3)
Women (%)	51.1	49.5	50.9	49.2	42.9	59.1	50.8	36.5
Obese (%)	23.9	23.8	16.8	19.6	28.5	20.6	16.8	23.6
Primary school or less (%)	65.3	51.7	41.8	49.4	74.2	54.4	38.0	56.0
Never smoker (%)	49.8	39.6	42.8	44.6	51.9	50.3	38.6	40.4
Physically Inactive (METS hour/week) (%)	44.5	43.1	36.5	39.6	45.9	40.7	36.6	42.9
Family history of colorectal cancer (%)	9.5	8.9	8.1	8.1	5.7	8.2	7.2	8.5
Family history of gastric cancer (%)	6.7	5.7	5.9	5.7	7.1	6.8	5.4	5.3
Night shift work history (%)	16.7	17.1	16.4	15.6	20.7	16.2	16.6	17.8
Retired (%)	50.0	45.5	38.4	52.8	65.2	41.1	36.2	60.5
**Diet habits; mean (SD)**								
Total energy intake (kcal/day)	1850 (744)	1872 (586)	1941 (663)	1873 (583)	1927 (654)	1856 (629)	1914 (628)	1941 (653)
Past alcohol consumption (g ethanol /day)	16.6 (27.9)	17.4 (28.1)	17.1 (25.3)	16.8 (26.2)	20.4 (30.8)	13.4 (22.9)	17.1 (26.3)	22.0 (30.4)
All red meat consumption (g/day)	57.9 (38.3)	61.4 (35.9)	65.0 (39.5)	62.2 (41.2)	57.3 (35.4)	59.2 (37.6)	66.8 (39.9)	63.3 (39.9)
Fruit consumption (g/day)	352 (218)	340 (205)	346 (234)	357 (210)	348 (209)	359 (216)	325 (205)	349 (224)
Vegetable consumption (g/day)	195 (141)	183 (105)	188 (113)	188 (123)	207 (156)	192 (129)	193 (128)	186 (113)
**Sleep characteristics**								
Sleep duration (mean; SD)		–	–	–	–	7.0 (1.4)	6.9 (1.2)	7.1 (1.4)
Daytime napping (%)	53.4	54.6	54.8	56.2	51.4			
Daytime napping frequency (days/week); (mean; SD)^a^	5.9 (1.9)	5.8 (2.0)	5.4 (2.2)	5.9 (12.0)	6.4 (1.5)	–	2.7 (1.3)	6.9 (0.2)
Daytime napping duration (minutes/day); (median; iqr)^a^	21 (49)	20 (41)	20 (24)	30 (45)	30 (45)	–	30 (45)	30 (45)
Ever sleep problems (%)	67.4	40.9	29.6	27.5	28.5	37.2	35.0	35.3
Sleep medication (%)	37.2	22.9	17.3	17.7	17.4	21.7	19.2	20.8
Frequent changes in bed time (%)	23.3	19.5	17.4	16.1	18.9	18.5	19.2	18.1

^**a**^Computed among participants with reported daytime napping.

*BMI* body mass index, *METS* metabolic equivalents, *SD* standard deviation, *iqr* interquartile range.

**Figure 1:**
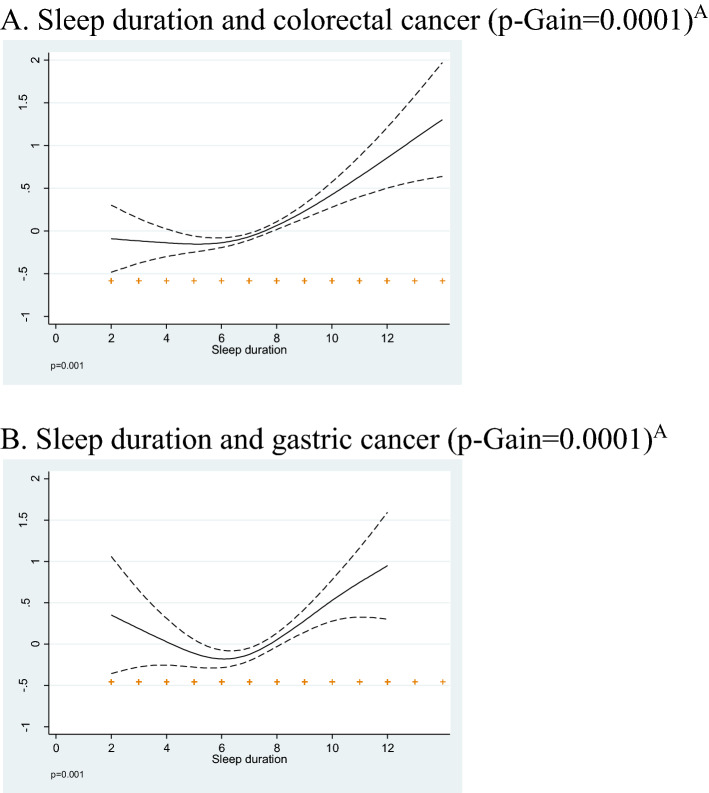
Multivariable adjusted General Additive Model (GAM) splines for the association (smooth function) of sleep duration and colorectal **(A)** and gastric **(B)** cancer risk in the MCC-Spain study. **(A)** Sleep duration and colorectal cancer (p-Gain = 0.0001). **(B)** Sleep duration and gastric cancer (p-Gain = 0.0001). Adjusted for age (continuous), centre (Barcelona, Madrid, Leon, Navarra, Cantabria, Guipuzcoa, Valencia, Huelva, Asturias, Granada, Murcia),, sex (female, male), and educational level (less than primary, primary, high school, university), family history of colorectal cancer or gastric cancer in first degree relatives (yes/no), body mass index (< 22.5, 22.5–24.9, 25–29.9, ≥ 30), leisure time physical activity (inactive, little active, moderately active, very active), smoking status (never, ex-smoker, current smoker).

We found a U-shape association between sleep duration and gastric cancer as shown in the multivariable adjusted GAM spline (Fig. 1). A similar but less pronounced shape of association was observed for CRC. The quadratic term of sleep duration was statistically significant (p < 0.001) for both colorectal and gastric cancer risk, showing departure from linearity. Associations of sleep duration categories, daytime naps with incident colorectal and gastric cancer are presented in Table 3. In multivariable adjusted models sleep duration of 8 hours and ≥ 9 hours were associated with an increase in the odds of CRC (OR_8 hours_: 1.27; 95% CI 1.08–1.49, OR_≥9 hours_: 1.59; 1.30–1.94) and gastric cancer (OR_8 hours_: 1.46; 95% CI 1.09–1.97, OR_≥9 hours_: 1.95; 1.35–2.76), compared to 7 h of sleep. Short sleep (≤ 5 hours) was associated with non-significant increase in the odds of gastric cancer (OR_≤5 hours_: 1.32; 95% CI; 0.93–1.88). Effects of short sleep on gastric cancer became stronger and statistically significant in full case-analyses among participants with diet information after additionally adjusting for diet, sleep, and shift work history (Supplemental Table 1). Daytime napping frequency and duration were both significantly associated with higher odds of colorectal and gastric cancer, compared to those who did not nap (Table 3). Cancer odds were highest among those who reported napping frequently (CRC: OR_6-7 days/week_: 1.24; 95% CI 1.09–1.42; gastric: OR_6-7 days/week_ 1.31; 95%CI 1.04–1.67) or longer (CRC: OR_>60 min_: 1.32; 95% CI 1.12–1.55; gastric: OR_>60 min_ 1.60; 95%CI 1.21–2.11). Mutually adjusting sleep duration for napping and vice-versa had minimal impact on the respective risk estimates (not shown). In combined analyses of napping frequency/duration the odds of cancer significantly increased among participants that reported frequent (6–7 naps/week) and longer (30 min) naps (CRC: OR_6-7 days/week_, _≥30 min_: 1.32; 95% CI 1.14–1.54; gastric: OR_6-7 days/week_, _≥30 min_: 1.56, 95%CI 1.21–2.02) and not for the other combinations of frequency and duration. The effects of long sleep duration and daytime napping on both tumors remained statistically significant and became stronger in full case-analysis among participants with diet information and after several additional adjustments for diet factors, sleep characteristics, and night shift work history (Supplemental Table 1). Sleep problems, duration of sleep problems (years) and frequent changes in time of sleep were not associated with colorectal or gastric cancer (Supplemental Table 2). In stratified analysis by BMI categories, long sleep was associated with a larger increase in CRC odds among individuals with BMI < 25 (colorectal: OR_≥9 hours_: 2.21, 1.55–3.16) than among overweight (BMI = 25–30: OR_≥9 hours_ 1.38; 1.01–1.87) and obese (BMI > 30: OR_≥9 hours_: 1.40; 0.93–2.09) participants (Table 4). We found no important variation in CRC risk estimates for daytime napping across BMI strata. On the contrary, the strongest effects on gastric cancer of extreme sleep durations and daytime napping were observed among overweight (BMI: 25–30) participants (p-for-interaction with sleep duration = 0.002).

**Table 3:** Sleep duration and daytime napping in relation to colorectal and gastric cancer odds in the MCC-Spain study.

Sleep duration (hours)	Colorectal cancer (N = 5606)						Gastric cancer (N = 3551)					
	Controls (N = 3598)		Cases (N = 2008)		OR [CI 95%]^a^	OR [CI 95%]^b^	controls (N = 3099)		Cases (N = 452)		OR [CI 95%]^a^	OR [CI 95%]^b^
	(n)	%	(n )	%			(n)	%	(n )	%		
≤ 5	472	13.1	259	12.9	1.05 (0.87, 1.28)	1.05 (0.86, 1.28)	419	13.5	68	15.0	1.29 (0.91, 1.83)	1.32 (0.93, 1.88)
6	723	20.1	337	16.8	0.98 (0.82, 1.17)	0.97 (0.81, 1.17)	624	20.1	70	15.5	0.99 (0.71, 1.39)	0.95 (0.68, 1.34)
7	1048	29.1	457	22.8	1.00 (Ref)	1.00 (Ref)	876	28.3	92	20.3	1.00 (Ref)	1.00 (Ref)
8	987	27.4	601	29.9	1.26 (1.08, 1.47)	1.27 (1.08, 1.49)	852	27.5	136	30.1	1.40 (1.05, 1.87)	1.46 (1.09, 1.97)
≥ 9	368	10.2	354	17.6	1.56 (1.29, 1.90)	1.59 (1.30, 1.94)	328	10.6	86	19.0	1.88 (1.33, 2.64)	1.95 (1.37, 2.76)

Daytime napping frequency (days/week)	Controls (N = 3495)		Cases (N = 1966)				Controls (N = 3044)		Cases (N = 444)			
No naps	1533	43.9	738	37.5	1.00 (Ref)	1.00 (Ref)	1323	43.5	174	39.2	1.00 (Ref)	1.00 (Ref)
1–2	302	8.6	128	6.5	1.02 (0.81, 1.30)	1.00 (0.79, 1.28)	236	7.8	32	7.2	1.25 (0.82, 1.92)	1.20 (0.78, 1.85)
3–5	253	7.3	139	7.1	1.13 (0.89, 1.42)	1.15 (0.91, 1.46)	203	6.7	25	5.6	0.96 (0.61, 1.52)	0.99 (0.62, 1.58)
6–7	1407	40.3	961	48.9	1.25 (1.10, 1.43)	1.24 (1.09, 1.42)	1281	42.1	213	48.0	1.26 (1.00, 1.58)	1.31 (1.04, 1.67)
Frequency (cont; per day/week)					1.03 (1.02, 1.05)	1.03 (1.01, 1.05)					1.03 (1.00, 1.06)	1.04 (1.01, 1.07)

Daytime napping duration (minutes/day)	Controls (N = 3437)		Cases (N = 1851)				Controls (N = 2987)		Cases (N = 441)			
No naps	1533	44.6	738	39.9	1.00 (Ref)	1.00 (Ref)	1323	44.3	174	39.5	1.00 (Ref)	1.00 (Ref)
< 15	269	7.8	103	5.6	0.79 (0.62, 1.02)	0.81 (0.63, 1.05)	226	7.6	28	6.3	0.97 (0.63, 1.50)	1.07 (0.69, 1.67)
15–29	432	12.6	230	12.4	1.06 (0.88, 1.28)	1.05 (0.86, 1.27)	372	12.4	43	9.8	0.90 (0.63, 1.29)	0.94 (0.65, 1.36)
30–60	546	15.9	321	17.3	1.19 (1.00, 1.41)	1.19 (1.00, 1.42)	490	16.4	82	18.6	1.33 (0.99, 1.79)	1.36 (1.00, 1.84)
> 60	657	19.1	459	24.8	1.30 (1.11, 1.53)	1.32 (1.12, 1.55)	575	19.3	114	25.9	1.53 (1.17, 2.00)	1.60 (1.21, 2.11)
Duration (cont; per 30 min napping)					*1.11 (1.06, 1.17)*	*1.12 (1.06, 1.18)*					*1.14 (1.05, 1.25)*	*1.14 (1.04, 1.24)*
**Daytime napping frequency and duration combined**												
No naps	1533	44.6	738	39.9	1.00 (Ref)	1.00 (Ref)	1323	44.3	174	39.5	1.00 (Ref)	1.00 (Ref)
1–5 naps/week, < 30 min	193	5.6	81	4.4	0.95 (0.71, 1.26)	0.95 (0.71, 1.27)	147	4.9	13	3.0	0.79 (0.43, 1.44)	0.83 (0.45, 1.53)
6–7 naps/week, < 30 min	508	14.8	252	13.6	0.97 (0.81, 1.16)	0.97 (0.80, 1.16)	451	15.1	58	13.2	0.97 (0.70, 1.35)	1.00 (0.74, 1.45)
1–5 naps/week, ≥ 30 min	350	10.2	166	9.0	1.08 (0.87, 1.35)	1.09 (0.88, 1.36)	280	9.4	44	10.0	1.31 (0.91, 1.91)	1.29 (0.88, 1.89)
6–7 naps/week, ≥ 30 min	853	24.8	614	33.2	1.32 (1.14, 1.53)	1.32 (1.14, 1.54)	785	26.3	152	34.5	1.48 (1.15, 1.91)	1.56 (1.21, 2.02)

^a^OR adjusted for age (continuous), centre (Barcelona, Madrid, Leon, Navarra, Cantabria, Guipuzcoa, Valencia, Huelva, Asturias, Granada, Murcia), sex (female, male), and educational level (less than primary, primary, high school, university).

^b^OR additionally adjusted for family history of colorectal cancer or gastric cancer in first degree relatives (yes/no), body mass index (< 22.5, 22.5–24.9, 25–29.9, ≥ 30), leisure time physical activity (inactive, little active, moderately active, very active), smoking status (never, ex-smoker, current smoker) and current occupational status (employed, unemployed, housewife, retired).

**Table 4:** Sleep duration and daytime napping in relation to colorectal and gastric cancer odds in the MCC-Spain study by BMI categories.

BMI categories	Colorectal cancer^a^					Gastric cancer^b^				
	Controls (N = 3598)		Cases (N = 2008)		OR [CI 95%]^c^	Controls (N = 3099)		Cases (N = 452)		OR [CI 95%]^c^
	(n)	%	(n )	%		(n)	%	(n )	%	
**BMI < 25**										
**Sleep duration (hours)**										
≤ 5	165	12.3	67	11.0	0.96 (0.65, 1.35)	138	12.8	15	11.7	0.72 (0.36, 1.45)
6	242	18.0	103	17.1	1.18 (0.85, 1.62)	197	18.2	26	20.3	1.19 (0.66, 2.14)
7	428	31.8	137	22.5	1.00 (Ref)	335	31.0	33	25.8	1.00 (Ref)
8	395	29.4	186	30.6	1.24 (0.93, 1.64)	322	29.8	30	23.4	0.83 (0.47, 1.47)
≥ 9	116	8.6	114	18.8	2.21 (1.55, 3.16)	90	8.3	24	18.8	1.95 (1.01, 3.74)
**Daytime napping frequency and duration**										
No naps	625	48.7	243	41.1	1.00 (Ref)	517	49.7	60	47.2	1.00 (Ref)
1–5 naps/week, < 30 min	89	6.9	33	5.9	1.09 (0.69, 1.71)	63	6.1	6	4.7	0.81 (0.30, 2.16)
6–7 naps/week, < 30 min	167	13.0	70	12.5	1.04 (0.75, 1.46)	142	13.6	17	13.4	1.15 (0.62, 2.11)
1–5 naps/week, ≥ 30 min	146	11.4	49	8.8	0.93 (0.63, 1.36)	112	10.8	14	11.0	1.01 (0.51, 2.00)
6–7 naps/week, ≥ 30 min	256	20.0	164	29.3	1.34 (1.02, 1.77)	207	19.9	30	23.6	1.33 (0.78, 2.24)
**BMI = 25–30**										
**Sleep duration (hours)**										
≤ 5	194	13.0	111	12.5	0.97 (0.71, 1.31)	177	13.4	32	15.4	1.92 (1.11, 3.32)
6	309	20.7	142	16.0	0.86 (0.65, 1.13)	267	20.2	31	14.9	1.30 (0.77, 2.23)
7	444	29.8	221	24.9	1.00 (Ref)	385	29.1	33	15.9	1.00 (Ref)
8	399	26.7	272	30.6	1.25 (0.99, 1.59)	355	26.8	68	32.7	2.19 (1.46, 3.66)
≥ 9	147	9.8	143	16.1	1.38 (1.01, 1.87)	137	10.4	44	21.2	3.16 (1.85, 5.38)
**Daytime napping frequency and duration**										
No naps	592	41.6	323	36.9	1.00 (Ref)	526	41.5	75	36.9	1.00 (Ref)
1–5 naps/week, < 30 min	73	5.1	28	3.4	0.69 (0.42, 1.12)	62	4.9	4	2.0	0.57 (0.20, 1.66)
6–7 naps/week, < 30 min	246	17.3	117	14.2	0.86 (0.65, 1.13)	216	17.0	26	13.0	0.88 (0.53, 1.45)
1–5 naps/week, ≥ 30 min	142	10.0	68	8.2	1.02 (0.72, 1.44)	113	8.9	17	8.5	1.20 (0.66, 2.18)
6–7 naps/week, ≥ 30 min	371	26.0	289	35.0	1.40 (1.12, 1.76)	351	27.7	78	39.0	**1.77 (1.21, 2.59)**
**BMI > 30**										
**Sleep duration (hours)**										
≤ 5	113	14.9	81	15.8	1.20 (0.80, 1.80)	99	14.9	21	18.1	1.03 (0.51, 2.05)
6	172	22.7	92	18.0	0.93 (0.64, 1.36)	156	23.5	13	11.2	0.45 (0.21, 0.95)
7	176	23.2	99	19.3	1.00 (Ref)	148	22.3	26	22.4	1.00 (Ref)
8	193	25.4	143	27.9	1.31 (0.92, 1.86)	168	25.3	38	32.8	1.17 (0.64, 2.12)
≥ 9	105	13.8	97	18.9	1.40 (0.93, 2.09)	94	14.1	18	15.5	1.06 (0.51, 2.20)
**Daytime napping frequency and duration**										
No naps	316	43.3	172	36.8	1.00 (Ref)	272	42.1	39	34.2	1.00 (Ref)
1–5 naps/week, < 30 min	31	4.2	20	4.3	1.22 (0.65, 2.30)	22	3.4	3	2.6	1.04 (0.27, 3.94)
6–7 naps/week, < 30 min	95	13.0	65	13.9	1.11 (0.75, 1.65)	88	13.6	15	13.2	1.39 (0.69, 2.81)
1–5 naps/week, ≥ 30 min	62	8.5	49	10.5	1.53 (0.96, 2.43)	54	8.3	13	11.4	1.99 (0.91, 4.32)
6–7 naps/week, ≥ 30 min	226	31.0	161	34.5	1.20 (0.88, 1.63)	212	32.7	44	38.6	1.60 (0.95, 2.71)

^a^p-for interaction (sleep duration) = 0.25, p-for-interaction (daytime napping) = 0.28.

^b^p-for interaction(sleep duration) = 0.002, p-for-interaction(daytime napping) = 0.70.

^c^OR adjusted for age (continuous), sex, centre (Barcelona, Madrid, Leon, Navarra, Cantabria, Guipuzcoa, Valencia, Huelva, Asturias, Granada, Murcia), sex (female, male), educational level (less than primary, primary, high school, university), family history of colorectal cancer or gastric cancer in first degree relatives (yes/no), body mass index (continuous), leisure time physical activity (inactive, little active, moderately active, very active), smoking status (never, ex-smoker, current smoker) and current occupational status (employed, unemployed, housewife, retired).

In stratified analysis by lifetime night shift work history, short sleep was associated with a significantly increased odds of colorectal (OR_≤5 hours:_ 1.69, 95%CI 1.04–2.74) and gastric cancer (OR_≤5 hours_: 2.75; 95%CI 1.15–6.59) among participants with night shift work history (LRT p-for interaction = 0.11) (Table 5). Long sleep duration (8 h and ≥ 9 h) was associated with higher odds of colorectal and gastric cancer of similar magnitude, compared to 7 h of sleep, in both strata. Regular long naps (6–7 days/week, ≥ 30 min) were associated with a greater increase in the odds of colorectal and gastric cancer among participants with night shift work history (CRC: OR_6-7 days/week, ≥30 min_ 1.75, 95%CI 1.23, 2.51; gastric: OR_6-7 days/week, ≥30 min_ 2.54, 95%CI 1.06–6.06), while a weaker or no association was described among participants with no shift work history (CRC: OR_6-7 days/week, ≥30min_1.33, 95%CI 1.11–1.61; gastric: OR_6-7 days/week_, ≥ 30 min 1.19; 0.87–1.63), although the statistical interaction between daytime naps and night work history was not significant.

**Table 5:** Sleep duration and daytime napping in relation to colorectal and gastric cancer odds in the MCC-Spain study by night shift work history.

Night shift work history	Colorectal cancer^a^					Gastric cancer^b^				
Never night shift work	Controls (N = 2557)		Cases (N = 1262)		OR [CI 95%]^c^	Controls (N = 2180)		Cases (N = 305)		OR [CI 95%]^c^
	(n)	%	(n )	%		(n)	%	(n )	%	
**Sleep duration (hours)**										
≤ 5	326	12.8	153	12.1	0.98 (0.76, 1.25)	292	13.4	41	13.4	1.11 (0.73, 1.71)
6	518	20.3	211	16.7	0.95 (0.76, 1.18)	442	20.3	47	15.4	0.85 (0.57, 1.28)
7	796	31.1	318	25.2	1.00 (Ref)	660	30.3	74	24.3	1.00 (Ref)
8	701	27.4	387	30.7	1.27 (1.05, 1.54)	602	27.6	95	31.2	1.43 (1.02, 2.01)
≥ 9	216	8.5	193	15.3	1.76 (1.37, 2.27)	184	8.4	48	15.7	2.22 (1.44, 3.43)
**Daytime napping frequency and duration combined**										
No naps	1072	43.3	454	37.0	1.00 (Ref)	910	42.6	124	41.6	1.00 (Ref)
1–5 naps/week, < 30 min	150	6.1	60	4.9	0.94 (0.67, 1.31)	116	5.4	9	3.0	0.64 (0.31, 1.32)
6–7 naps/week, < 30 min	371	15.0	174	14.2	0.96 (0.77, 1.20)	327	15.3	39	13.1	0.86 (0.57, 1.28)
1–5 naps/week, ≥ 30 min	273	11.0	127	10.4	1.14 (0.88, 1.47)	218	10.2	31	10.4	1.00 (0.64,1.57)
6–7 naps/week, ≥ 30 min	610	24.6	411	33.5	1.33 (1.11, 1.61)	564	26.4	95	31.9	1.19 (0.87, 1.63)

Ever night shift work	Controls (N = 605)		Cases (N = 339)			Controls (N = 503)		Cases (N = 88)		
**Sleep duration (hours)**										
≤ 5	79	13.1	52	15.3	1.69 (1.04, 2.74)	65	12.9	18	20.5	2.75 (1.15, 6.59)
6	124	20.5	58	17.1	1.16 (0.73, 1.83)	102	20.3	13	14.8	1.40 (0.60, 3.43)
7	172	28.4	68	20.1	1.00 (ref)	140	27.8	12	13.6	1.00 (Ref)
8	154	25.5	102	30.1	1.60 (1.06, 2.41)	128	25.5	26	29.6	2.27 (1.01, 5.11)
≥ 9	76	12.6	59	17.4	1.54 (0.95, 2.51)	68	13.5	19	21.6	2.53 (1.06, 6.06)
**Daytime napping frequency and duration combined**										
No naps	249	42.2	106	31.6	1.00 (Ref)	216	43.6	26	30.2	1.00 (Ref)
1–5 naps/week, < 30 min	32	5.4	15	4.5	1.40 (0.68, 2.85)	25	5.0	4	4.7	1.52 (0.43, 5.36)
6–7 naps/week, < 30 min	80	13.6	48	14.3	1.27 (0.80, 2.02)	75	15.2	10	11.6	1.31 (0.54, 3.16)
1–5 naps/week, ≥ 30 min	60	10.2	27	8.0	1.21 (0.70, 2.10)	43	8.7	9	10.5	3.25 (1.22, 8.68)
6–7 naps/week, ≥ 30 min	169	28.6	140	41.7	1.75 (1.23, 2.51)	136	27.5	37	43.0	2.97 (1.54, 5.72)

^a^p-for interaction (sleep duration) = 0.21, p-for-interaction(daytime napping) = 0.77.

^b^p-for interaction(sleep duration) = 0.30, p-for-interaction(daytime napping) = 0.38.

^c^OR adjusted for age (continuous), sex (male, female), centre (Barcelona, Madrid, Leon, Navarra, Cantabria, Guipuzcoa, Valencia, Huelva, Asturias, Granada, Murcia),, educational level (less than primary, primary, high school, university), family history of colorectal cancer or gastric cancer in first degree relatives (yes/no), body mass index (< 22.5, 22.5–24.9, 25–29.9, ≥ 30), leisure time physical activity (inactive, little active, moderately active, very active), smoking status (never, ex-smoker, current smoker) and current occupational status (employed, unemployed, housewife, retired).

In stratified analyses by sex, the association between sleep duration and cancer varied slightly across sexes (opposite direction for short and long sleep), but there was no evidence for a statistically significant sex-interaction with sleep duration (Supplemental Table 3). Women in the top category of napping frequency and duration had a higher odds for gastric cancer than men (LRT p-for-interaction = 0.03). In analyses stratified by education associations of sleep duration and cancer were observed across both strata of education (primary or less/high school or more) (Supplemental Table 4). A finer analysis using 4 strata revealed a similar risk pattern for sleep duration (data not shown); results for 9 + hours of sleep were insignificant and weaker among those with the lowest education (less than primary) compared to the rest of the groups. Daytime napping results were stronger in the stratum with lowest education for CRC but results for gastric cancer were similar across strata of education. In stratified analysis by age at diagnosis/interview, the effects of longer sleep duration and daytime napping on colorectal and gastric cancer were more pronounced among participants younger than 50 years than among those older than 50 years, but no statistically significant interaction was observed (Supplemental Table 5). Results for colorectal cancer showed no significant heterogeneity by TNM staging or anatomical site (Supplemental Table 6). Associations with long sleep duration and napping were a bit stronger for stage IV colorectal cancer but also strong and significant for stage 0–II colorectal tumors. Most of the associations were retained in analyses by anatomical cancer site and Lauren’s classification categories for gastric cancer (Supplemental Table 7). Long sleep duration was associated with an increased odds for both tumors located in the colon and rectum, while most of the risk estimates did not differ between non-cardia and cardia/esophageal cancer. In sensitivity analyses excluding participants with report of sleep problems in the 5 years prior to recruitment, results were unchanged (Supplemental Table 8). After the exclusion of retired participants and participants with more than 6 months between the date of diagnosis and interview most results were unchanged or became stronger (data not shown).

## Discussion

In this large case–control study we found that participants with longer sleep duration (8 hours and ≥ 9 hours) had significantly increased odds of colorectal and gastric cancer, compared to those with 7 hours of sleep. Short sleep duration (≤ 5 hours) was also associated with increased, but not statistically significant, gastric cancer odds. Frequent (6–7 days/week) long (> 60 min) naps were associated with increased odds of colorectal and gastric cancer. The effects of short sleep duration and napping were stronger among participants with night shift work history for both tumors.

Long sleep duration was independently associated with colorectal and gastric cancer in our study. Long sleep duration has been associated with higher all-cause and cancer-specific mortality in several meta-analyses including a large number of observational studies^4,23,24^. To date, only a few studies have examined the association of sleep duration with cancer risk for tumors other than breast cancer^11,14^. Our findings for an association of long sleep with CRC are in line with results from two prospective US cohorts of health professionals previously reported an increased risk of CRC among participants with sleep duration of 8 hours and ≥ 9 hours (compared to 7 hours of sleep), especially among individuals who were overweight or snored regularly^16^. Although we lacked information on snoring or sleep disordered breathing, our results did not change after adjusting for BMI/obesity and impaired sleep quality, two surrogates of sleep apnoea. Long sleep exhibited a stronger effect on CRC among individuals with a normal BMI. This may indicate that mechanisms other than increased adiposity—a well-known risk factor for CRC and gastric cancer—might be involved in the potential link between long sleep and cancer. A 40% increase in risk of CRC was associated with sleep durations of 10 + hours/day in a cohort of female teachers^25^. Our study extends these findings to both sexes and to the general population across occupational groups. Both long (≥ 9 hours) and short (≤ 5 hours) sleep have been associated with an increase in the incidence of CRC among postmenopausal women^17^. In our study, short sleep (≤ 5 hours) was associated with borderline higher odds of gastric cancer, but not CRC. Previous studies have identified associations of short sleep with all-cause mortality, cancer-specific mortality and cancer incidence^12^. In line with our results, a US cohort of older adults (the NIH-AARP Diet and Health Study) reported a significantly increased gastric cancer risk in male short (5–6 hours) sleepers^18^. Our findings for short sleep duration and gastric cancer are also supported by a recent Mendelian Randomization study that analysed 22-site specific cancers among UK Biobank participants and reported suggestive associations of genetic liability to short sleep duration with higher odds of several gastrointestinal cancers including gastric, pancreatic and CRC^26^.

Daytime napping frequency and duration were associated with increasing odds of colorectal and gastric cancer, with highest odds observed in participants with frequent (6–7 naps/week) and long (≥ 30 min) naps. When napping analyses were further adjusted for sleep duration and other sleep characteristics results were robust, indicating independent association with cancer. This is the first study to examine the role of daytime napping—commonly known as “siesta”—in relation to colorectal and gastric cancer risk. In a previous study no napping was associated with a greater all-cancer incidence in males, compared to < 30 min napping, but longer napping durations (30 + min) were also associated with a higher cancer risk^27^. Similarly in our data, gastrointestinal cancer odds increased among participants reporting longer naps (≥ 30 min) but not among those with shorter napping duration (< 30 min) independently of napping frequency. Interestingly, in a study including 4869 CRC survivors, prediagnostic napping was associated with higher total and cardiovascular disease-specific mortality^19^. The role of daytime napping and its frequency and duration in modifying the association of sleep needs to be explored in future studies of cancer and other chronic disease outcomes.

In stratified analysis by night shift work history we found a stronger effect of short sleep and napping among subjects with night shift work history, and this risk pattern was consistent for both colorectal and gastric cancer. Sleep disruption is one of the suggested mechanisms for the link between night shift work and cancer risk^28^. While in our previous analyses long-term night shift work has been shown to increase the odds of gastric and colorectal^20,21^, the present study suggests that short sleep duration and napping may interact with shift work with joint effects on gastrointestinal cancer risk. Although in our study, sleep problems could not be attributed to night shift work, our findings corroborate with a few other studies suggesting combined effects of long-term night shift work and long sleep duration on total cancer incidence and mortality^27^, breast cancer^29^ and lung cancer^30^. Night shift work leads to acute sleep loss and impaired sleep quality^31,32^, compensational daytime napping on work days and longer sleep on days off^6,32,33^. Shift work related sleep disturbances may become chronic and persist even after quitting shift work^34^, and in retirement^35^. Shift workers who are especially vulnerable to shift work schedules are more likely to develop “shift work sleep disorder” (SWD) which consists of chronic insomnia and/or daytime sleepiness^36^. However it is currently unknown if shift workers with SWD are at a greater risk of developing later chronic diseases compared to shift workers without SWD^37^. Our results suggest that participants with short sleep and daytime naps may be more susceptible to the negative effects of night work. Alternatively participants with night shift work history that develop sleep problems might be at a higher risk of colorectal and gastric cancer, compared to night workers without sleep complaints. Our finding of joint effects between circadian and sleep disruption is novel and needs to be confirmed in prospective cohort studies.

Animal and human studies suggest a genetic basis for sleep duration, thus, short and long sleepers may represent phenotypes of the function of clock genes in human sleep^38–40^. Experimental data provide a physiological basis for the inter-individual variability and the intra-individual stability of habitual sleep duration^38^. Sleep is well known for its contribution to the maintenance of the immune system and regulation of human metabolism^41,42^. Several mechanisms for the negative effects of long sleep on mortality have been suggested and discussed by Grander and Drummond^43^ and many of them may have implications for cancer outcomes: First, reported long sleep might reflect disturbances in sleep continuity or sleep architecture and thus may result from impaired sleep quality or fragmented sleep^44^. Second, long sleep has been associated with feelings of fatigue and lethargy which in turn may decrease resistance to stress and disease. Third, long sleep may influence the immune system and expression of cytokines^45–47^. Fourth long sleep may be associated with shorter photoperiod and inadequate light exposure patterns^48^. Fifth, long sleep has been consistently associated with a more sedentary behaviour, less healthy lifestyle and obesity^49–52^. Furthermore, long sleepers were found more likely to be divorced, living alone, unemployed, and have a lower socioeconomic status^50^. Sixth, depression or other underlying chronic disease such as sleep apnoea or diabetes that lead to longer sleep or longer time in bed may mediate the association between long sleep and cancer. Last, there is a possibility that poor health including cancer leads to long sleep rather than the opposite^43^. On the other hand, several biological mechanisms may explain the potential link between short sleep with cancer risk. Sleep deprivation has been associated with impaired immune function, elevated levels of inflammatory markers and deregulation of cortisol levels that may affect the tumor surveillance system^53–55^. Specifically for gastric cancer, a disrupted immune–inflammation balance might promote *H. pylori* related gastric carcinogenesis^41^. Sleep deficiency has been also linked to higher adiposity, metabolic syndrome and type II diabetes that may in turn further increase the risk of cancer^56–58^. Furthermore, short sleep duration might be related to increased light-at-night exposure and thus lower nocturnal melatonin levels^38^, a hormone with well-known direct and indirect oncostatic properties^59,60^. Finally, sleep loss and/or circadian misalignment may be associated with gut microbiota disruption and subsequent increased risk for metabolic disease and CRC^61,62^.

Our study has several limitations. First, reverse causality could partly explain some of the observed associations since cancer status may affect sleep duration and napping habits in both directions (towards more but also less sleep). Similar to most previous epidemiological studies, we assessed current sleep duration and assumed it to be representative of habitual and past sleep duration during adulthood within individuals, but likely reflects relatively recent exposures. In order to address this question, in a follow up of the MCC-Spain study additional sleep information was collected at age 40 years and in the year prior to recruitment/cancer diagnosis among breast and prostate cancer cases and respective controls. In preliminary analysis, we found a significant correlation between sleep durations at age 40 and in the year before diagnosis and results did not vary by case–control status (data not shown). These results are supportive of our assumption that current sleep duration assessed in late adulthood could be indicative of past sleep duration in mid adulthood. Furthermore, in our study only incident cases were recruited and interviews were scheduled soon after cancer diagnoses. Our results did not change when we excluded interviews that took place 6 months or later after cancer diagnoses (data not shown). In order to reduce the potential for reverse causality, we conducted sensitivity analyses excluding participants with sleep problems in the 5 years before diagnosis/recruitment, which did not materially change the results. In addition, we found no evidence for significant risk variation across colorectal cancer stages in TNM stage-stratified analyses. Second, self-reported sleep duration estimates may contain error and are in poor or moderate agreement with objective (polysomnography or actigraphy) measurements in some but not all studies^63–65^. Participants might have provided information on how many hours they spend in bed instead of actual sleep time, although these two metrics tend to correlate. However, report of current sleep duration is unlikely differential between cases and controls since no recall of past exposure was involved and sleep duration is not a well-known risk factor for cancer, thus, exposure misclassification may have biased our estimates to the null. Third, sleep duration was missing in 8.8% of CRC controls, 9% in gastric cancer controls, 6.1% of CRC cases and 1.5% in gastric cancer cases, but no differences were found in age, BMI, family history of cancer between participants with and without sleep duration information. Missingness in sleep duration was associated with education and lifestyle (opposite pattern for cases and for controls) but in stratified analyses by education most results were robust. Fourth, we assessed habitual sleep duration once and did not record changes or sustained effects of sleep duration over time, although we controlled for frequent changes of sleeping time that could account for night-to-night variability in sleep duration. Epidemiology is still limited due to constraints in measuring sleep with existing questionnaires^66^, and future studies should include detailed sleep assessment over critical periods in life that would help reconstruct and evaluate a person’s “lifetime sleep history”^67^. Fifth, although our study was large, power was limited in stratified analyses, especially for gastric cancer. Sixth, although our study extensively controlled for potential confounders, residual confounding cannot be entirely ruled out. In addition, psychiatric conditions such as depression or anxiety were not assessed and thus not controlled for analytically. Last, similar to other population-based case–control studies low to moderate response rates were observed in both cases and controls, especially among gastric cancer patients due to poor disease prognosis. It is possible that gastric cancer patients with more advanced disease at diagnosis were underrepresented in our study. The reasons for the relatively low response rates may have differed between cases and controls and could have led to selection bias. Participants with a higher socioeconomic or educational status were more likely to participate in the study, especially among controls. However, we found no clear differences of the effects according to groups of education, where selection bias potential would be minimized.

Strengths of this study include the large sample size with enough participants in the categories of extreme sleep duration (29% reported ≤ 5 h and 10% reported ≥ 9 h), the collection of information on a wide range of known and suspected risk factors for both tumors and extensive control for potential confounders, including other sleep characteristics such as sleep quality and sleep timing, to account for potential confounding due to impaired sleep. Most results were strong and consistent between colorectal and gastric cancer and remained significant after additional adjustments and in several stratified and sensitivity analyses, suggesting that our findings were unlikely entirely due to chance.

In this large population-based case–control study we described an association of long sleep duration (8 hours and ≥ 9 hours) with increased odds of colorectal and gastric cancer. Short sleep duration (≤ 5 hours) was associated with increased—but not statistically significant—odds of gastric cancer. Short sleep and daytime napping, especially frequent (6–7 days/week) long (≥ 30 min) naps, were associated with increased colorectal and gastric cancer odds, in particular among participants with night shift work history. Our findings support a potential role of extreme nighttime sleep duration and daytime napping in the odds of colorectal and gastric cancer and suggest possible joint effects of sleep and circadian disruption on the risk of gastrointestinal cancer.

## Supplementary Information

Supplementary Tables.
