# Supplementary material for: Sleep duration and napping in relation to colorectal and gastric cancer in the MCC-Spain study
Source: Sci Rep. 2021 Jun 3;11:11822. doi: 10.1038/s41598-021-91275-3 (PMC8175745; doi:10.1038/s41598-021-91275-3)
Supplement: Supplementary file 1 — Supplementary Tables. [file 41598_2021_91275_MOESM1_ESM.docx]

**ORIGINAL ARTICLE – SUPPLEMENTAL TABLES**

**Sleep duration and napping in relation to colorectal and gastric cancer risk (MCC-Spain study)**

Kyriaki Papantoniou^1,2^, Gemma Castaño-Vinyals^2,3,4,5^, Ana Espinosa^2,3,4,5^, Michelle C Turner^2,3,4,6^, Vicente Martín-Sánchez^4,7^, Delphine Casabonne^4,8^, Nuria Aragonés^4,9^, Inés Gómez-Acebo^4,10,11^, Eva Ardanaz^4,12^, Jose-Juan Jimenez Moleon^4,13,14^, Pilar Amiano^4,15,16^, Ana Molina-Barceló^17^, Juan Alguacil^4, 18^, Guillermo Fernández-Tardón^4,19^ , José María Huerta^4,20^, Natalia Hernández-Segura^7^, Beatriz Perez-Gomez^4,21,22^, Javier Llorca^4,10^, Juana Vidán Alli^4,12^, Rocıo Olmedo-Requena^4,13,14^, Leire Gil^4,15,16^, Carmen Castañon López^23^, Marina Pollan^4,21^, Manolis Kogevinas^2,3,4,5^,Victor Moreno^4,24,25,26^

1. Department of Epidemiology, Center of Public Health, Medical University of Vienna, Vienna, Austria
2. ISGlobal, Barcelona, Spain
3. Universitat Pompeu Fabra (UPF), Barcelona, Spain
4. Consortium for Biomedical Research in Epidemiology and Public Health (CIBERESP), Madrid, Spain
5. IMIM (Hospital del Mar Medical Research Institute), Barcelona, Spain
6. McLaughlin Centre for Population Health Risk Assessment, University of Ottawa, Ottawa, Ontario, Canada
7. Biomedicine Institue (IBIOMED), University of León, Leon, Spain
8. Unit of Molecular and Genetic Epidemiology in Infections and Cancer (UNIC-Molecular), Cancer Epidemiology Research Programme, IDIBELL, Catalan Institute of Oncology, Hospitalet De Llobregat, Spain
9. Epidemiology Section, Public Health Division, Department of Health of Madrid, 28035 Madrid, Spain​
10. University of Cantabria, Santander, Spain
11. IDIVAL, Santander, Spain
12. Navarra Public Health Institute, Pamplona, Spain; IdiSNA, Navarra Institute for Health Research, Pamplona, Spain
13. Department of Preventive Medicine and Public Health, University of Granada, Spain
14. Instituto de Investigacion Biosanitaria de Granada (ibs.GRANADA), Hospitales Universitarios de Granada/Universidad de Granada, Granada, Spain
15. Public Health Division of Gipuzkoa, San Sebastian, Spain
16. Biodonostia Research Institute, San Sebastian, Spain
17. Cancer and Public Health Area, FISABIO—Public Health, Valencia, Spain
18. Centro de Investigación en Salud y Medio Ambiente (CYSMA), Universidad de Huelva, Campus Universitario de El Carmen, 21071 Huelva, Spain
19. Health Research Institute of the Principality of Asturias, University of Oviedo, Oviedo, Spain
20. Department of Epidemiology, Murcia Regional Health Council, IMIB-Arrixaca, Murcia, Spain
21. Cancer Epidemiology Unit of the National Center for Epidemiology, Carlos III Institute of Health, Madrid, Spain
22. Cancer Epidemiology Research Group, Oncology and Hematology Area, IIS Puerta de Hierro (IDIPHIM), Majadahonda, Madrid, Spain
23. Servicio de Oncología. Complejo Asistencial Universitario de León, Leon, Spain
24. Unit of Biomarkers and Susceptibility, Oncology Data Analytics Program, Catalan Institute of Oncology (ICO). Hospitalet de Llobregat, Barcelona, Spain.
25. Colorectal Cancer Group, ONCOBELL Program, Bellvitge Biomedical Research Institute (IDIBELL), Hospitalet de Llobregat, Barcelona, Spain.
26. Department of Clinical Sciences, Faculty of Medicine, University of Barcelona, Barcelona, Spain

**Corresponding author:**

Kyriaki Papantoniou, kyriaki.papantoniou@meduniwien.ac.at

Department of Epidemiology, Center of Public Health, Medical University of Vienna

Kinderspitalgasse 15, 1090, Vienna, Austri

**Supplemental Table 1.** Full case analysis of sleep duration and daytime napping in relation to colorectal and gastric cancer risk among participants **with diet information and additional adjustment for potential confounders (N=5312).**

|  | **Colorectal cancer (N=4963)** | | | | **Gastric cancer (N=3079)** | | | |
| --- | --- | --- | --- | --- | --- | --- | --- | --- |
|  | **OR [CI 95%]^a^** | **OR [CI 95%]^b^** | **OR [CI 95%]^c^** | **OR [CI 95%]^d^** | **OR [CI 95%]^a^** | **OR [CI 95%]^b^** | **OR [CI 95%]^c^** | **OR [CI 95%]^d^** |
| **Sleep duration (hours)** |  |  |  |  |  |  |  |  |
| ≤5 | 1.10 (0.89, 1.36) | 1.10 (0.88, 1.38) | 1.08 (0.87, 1.35) | 1.14 (0.91, 1.42) | 1.47 (0.98, 2.11) | **1.55 (1.01, 2.40)** | **1.54 (1.02, 2.33)** | **1.55 (1.03, 2.35)** |
| 6 | 0.97 (0.80, 1.17) | 0.98 (0.80, 1.19) | 0.96 (0.79, 1.17) | 1.00 (0.82, 1.23) | 0.99 (0.67, 1.46) | 0.99 (0.67, 1.48) | 1.03 (0.70, 1.54) | 1.03 (0.65, 1.44) |
| 7 | 1.00 (Ref) | 1.00 (Ref) | 1.00 (Ref) | 1.00 (Ref) | 1.00 (Ref) | 1.00 (Ref) | 1.00 (Ref) | 1.00 (Ref) |
| 8 | **1.27 (1.07, 1.50)** | **1.27 (1.07, 1.51)** | **1.24 (1.04, 1.48)** | **1.32 (1.10, 1.58)** | **1.58 (1.13, 2.22)** | **1.54 (1.10, 2.17)** | **1.68 (1.19, 2.37)** | **1.63 (1.15, 2.31)** |
| ≥9 | **1.57 (1.27, 1.94)** | **1.55 (1.24, 1.93)** | **1.53 (1.23, 1.90)** | **1.66 (1.32, 2.08)** | **2.02 (1.35, 3.02)** | **1.97 (1.32, 2.95)** | **2.03 (1.35, 3.05)** | **2.10 (1.38, 3.20)** |
|  | **Colorectal cancer (N=4667)** | | | | **Gastric cancer (N=2967)** | | | |
| **Daytime napping frequency and duration combined** | **OR [CI 95%]^a^** | **OR [CI 95%]^b^** | **OR [CI 95%]^c^** | **OR [CI 95%]^d^** | **OR [CI 95%]^a^** | **OR [CI 95%]^b^** | **OR [CI 95%]^c^** | **OR [CI 95%]^d^** |
| **No naps** | **Ref** | **Ref** | **Ref** | **Ref** | **Ref** | **Ref** | **Ref** | **Ref** |
| 1-5 naps/week, <30 min | 0.88 (0.65, 1.21) | 0.92 (0.67, 1.25) | 0.87 (0.63, 1.19) | 0.92 (0.67, 1.25) | 0.87 (0.45, 1.52) | 0.92 (0.47, 1.79) | 0.81 (0.41, 1.59) | 0.83 (0.44, 1.67) |
| 6-7 naps/week, <30min | 0.97 (0.79, 1.18) | 0.97 (0.80, 1.18) | 0.97 (0.79, 1.19) | 1.02 (0.84, 1.25) | 1.00 (0.69, 1.45) | 0.98 (0.67, 1.43) | 0.97 (0.66, 1.41) | 0.91 (0.65, 1.37) |
| 1-5 naps/week, ≥30 min | 1.09 (0.86, 1.38) | 1.10 (0.87, 1.40) | 1.08 (0.85, 1.38) | 1.12 (0.88, 1.42) | 1.36 (0.89, 2.07) | 1.26 (0.82, 1.94) | 1.30 (0.84, 2.00) | 1.31 (0.95, 2.19) |
| 6-7 naps/week, ≥30 min | **1.27 (1.08, 1.49)** | **1.25 (1.06, 1.47)** | **1.25 (1.06, 1.48)** | **1.33 (1.13, 1.57)** | **1.39 (1.03, 1.86)** | **1.35 (1.00, 1.82)** | **1.34 (0.99, 1.81)** | 1.26 (0.93, 1.75) |

^a^OR adjusted for age (continuous), ), centre (Barcelona, Madrid, Leon, Navarra, Cantabria, Guipuzcoa, Valencia, Huelva, Asturias, Granada, Murcia), and educational level (less than primary, primary, high school, university), sex (female, male), family history of colorectal cancer or gastric cancer in first degree relatives (yes/no), body mass index (<22.5, 22.5-24.9, 25-29.9, ≥30), leisure time physical activity (inactive, little active, moderately active, very active), smoking status (never, ex-smoker, current smoker) and current occupational status (employed, unemployed, housewife, retired) in **full-case analysis among participants with diet information**.

^b^OR additionally adjusted for **sleep characteristics**: ORs for sleep duration additionally adjusted for daytime napping frequency and duration, ever sleep problems, frequent changes is bed time; ORs for daytime nap additionally adjusted for sleep duration, ever sleep problems and frequent changes in bedtime

^c^OR additionally adjusted for **diet characteristics**: past alcohol consumption (quartiles), total energy intake in grams/day (quartiles), red meat consumption in grams/day (quartiles), fruit consumption in grams/day (quartiles), vegetable consumption in grams/day (quartiles)

^d^OR additionally adjusted for **night** **shift work history** (never shift work, permanent night shifts, rotating night shifts, rotating shifts, housewives)

**Supplemental Table 2.** Sleep quality in relation to colorectal and gastric cancer risk in the MCC-Spain study

|  | **Colorectal cancer** | | | | | | **Gastric cancer** | | | | | |
| --- | --- | --- | --- | --- | --- | --- | --- | --- | --- | --- | --- | --- |
|  | **Controls** | | **Cases** | | **OR [CI 95%]^a^** | **OR [CI 95%]^b^** | **Controls** | | **Cases** | | **OR [CI 95%]^a^** | **OR [CI 95%]^b^** |
| **Ever sleep problems** |  |  |  |  |  |  |  |  |  |  |  |  |
| No | 2295 | 63.8 | 1329 | 66.3 | 1.00 (Ref) | 1.00 (Ref) | 2017 | 65.1 | 322 | 71.2 | 1.00 (Ref) | 1.00 (Ref) |
| Yes | 1300 | 36.2 | 674 | 33.7 | 0.94 (0.83, 1.06) | 0.94 (0.83, 1.06) | 1080 | 34.9 | 130 | 28.8 | 0.79 (0.63, 0.99) | 0.80 (0.64, 1.01) |
| **Duration of Sleep Problems (Years)** |  |  |  |  |  |  |  |  |  |  |  |  |
| <10 | 532 | 16.5 | 229 | 12.9 | 0.85 (0.71, 1.01) | 0.85 (0.71, 1.01) | 428 | 15.5 | 46 | 11 | 0.73 (0.52, 1.01) | 0.74 (0.52, 1.01) |
| 10-19 | 223 | 6.9 | 108 | 6.1 | 0.86 (0.67, 1.10) | 0.86 (0.67, 1.10) | 173 | 6.3 | 25 | 6 | 0.94 (0.59, 1.45) | 0.92 (0.59, 1.45) |
| >20 | 178 | 5.5 | 115 | 6.5 | 1.12 (0.86, 1.44) | 1.10 (0.85, 1.41) | 149 | 5.4 | 25 | 6 | 1.05 (0.66, 1.67) | 1.04 (0.67, 1.65) |
| **Frequent Changes in bedtime** |  |  |  |  |  |  |  |  |  |  |  |  |
| No | 2918 | 81.6 | 1606 | 80.7 | 1.00 (Ref) | 1.00 (Ref) | 2518 | 81.8 | 363 | 81.2 | 1.00 (Ref) | 1.00 (Ref) |
| Yes | 657 | 18.4 | 382 | 19.2 | 1.03 (0.88, 1.19) | 1.02 (0.87, 1.18) | 560 | 18.2 | 84 | 18.8 | 1.06 (0.81, 1.39) | 1.05 (0.79, 1.38) |

^a^OR adjusted for age (continuous), centre (Barcelona, Madrid, Leon, Navarra, Cantabria, Guipuzcoa, Valencia, Huelva, Asturias, Granada, Murcia), , sex (female, male), and educational level (less than primary, primary, high school, university);

^b^OR additionally adjusted for family history of colorectal cancer or gastric cancer in first degree relatives (yes/no), body mass index (<22.5, 22.5-24.9, 25-29.9, ≥30), leisure time physical activity (inactive, little active, moderately active, very active), smoking status (never, ex-smoker, current smoker) and current occupational status (employed, unemployed, housewife, retired).

**Supplemental Table 3.** Sleep duration and daytime napping in relation to colorectal and gastric cancer risk in the MCC-Spain study **by sex**.

|  | **Colorectal cancer*** | | | | | **Gastric cancer*** | | | | |
| --- | --- | --- | --- | --- | --- | --- | --- | --- | --- | --- |
| **MEN** | **Controls**  **(N=1822)** | | **Cases**  **(N=1280)** | | **OR [CI 95%]^a^** | **Controls**  **(N=1700)** | | **Cases**  **(N=303)** | | **OR [CI 95%]^a^** |
| **Sleep duration (hours)** | **(n)** | **%** | **(n)** | **%** |  | **(n)** | **%** | **(n )** | **%** |  |
| ≤5 | 231 | 12.7 | 148 | 11.6 | 1.04 (0.79, 1.35) | 220 | 12.9 | 40 | 13.2 | 1.19 (0.76, 1.87) |
| 6 | 365 | 20.0 | 210 | 16.4 | 0.94 (0.74, 1.19) | 338 | 19.9 | 46 | 15.2 | 0.84 (0.55, 1.28) |
| 7 | 515 | 28.3 | 291 | 22.7 | 1.00 (Ref) | 472 | 27.8 | 67 | 22.1 | 1.00 (Ref) |
| 8 | 501 | 27.5 | 388 | 30.3 | **1.28 (1.04, 1.57)** | 473 | 27.8 | 91 | 30.0 | 1.30 (0.91, 1.87) |
| ≥9 | 210 | 11.5 | 243 | 19.0 | **1.77 (1.37, 2.28)** | 197 | 11.6 | 59 | 19.5 | **2.12 (1.38, 1.87)** |
| **Daytime napping frequency and duration combined** |  |  |  |  |  |  |  |  |  |  |
| No naps | 623 | 34.2 | 379 | 30.2 | 1.00 (Ref) | 577 | 35.3 | 110 | 34.4 | 1.00 (Ref) |
| 1-5 naps/week, <30 min | 76 | 4.2 | 51 | 4.3 | 1.21 (0.81, 1.80) | 69 | 4.2 | 10 | 3.4 | 0.92 (0.44, 1.92) |
| 6-7 naps/week, <30min | 282 | 15.5 | 162 | 13.8 | 0.99 (0.77, 1.26) | 271 | 16.6 | 32 | 10.8 | 0.71 (0.46, 1.12) |
| 1-5 naps/week, ≥30 min | 188 | 10.3 | 109 | 9.3 | 0.99 (0.75, 1.32) | 162 | 9.9 | 31 | 10.5 | 1.14 (0.71, 1.82) |
| 6-7 naps/week, ≥30 min | 578 | 31.7 | 474 | 40.3 | **1.42 (1.18, 1.72)** | 554 | 33.9 | 112 | 38.1 | 1.37 (1.00, 1.89) |
| **WOMEN** | **Controls**  **(N=1776)** | | **Cases**  **(N=728)** | | **OR [CI 95%]^a^** | **Controls**  **(N=1700)** | | **Cases**  **(N=303)** | | **OR [CI 95%]^a^** |
| **Sleep duration (hours)** | **(n)** | **%** | **(n)** | **%** |  | **(n)** | **%** | **(n)** | **%** |  |
| ≤5 | 241 | 13.6 | 111 | 17.4 | 1.06 (0.78, 1.46) | 198 | 14.3 | 28 | 18.8 | 1.40 (0.76, 2.55) |
| 6 | 358 | 20.2 | 127 | 15.2 | 0.98 (0.73, 1.31) | 286 | 20.5 | 24 | 16.1 | 1.16 (0.63, 2.13) |
| 7 | 533 | 30.0 | 166 | 22.8 | 1.00 (Ref) | 399 | 28.9 | 25 | 16.8 | 1.00 (Ref) |
| 8 | 486 | 27.4 | 213 | 29.3 | 1.26 (0.97, 1.63) | 376 | 27.1 | 45 | 30.2 | 1.67 (0.98, 2.84) |
| ≥9 | 158 | 8.9 | 111 | 15.2 | **1.40 (1.00, 1.96)** | 139 | 9.4 | 27 | 18.1 | 1.55 (0.82, 2.92) |
| **Daytime napping frequency and duration combined** |  |  |  |  |  |  |  |  |  |  |
| No naps | 910 | 53.9 | 359 | 53.1 | 1.00 (Ref) | 746 | 54.5 | 64 | 43.8 | 1.00 (Ref) |
| 1-5 naps/week, <30 min | 117 | 6.9 | 30 | 4.4 | 0.74 (0.47, 1.17) | 78 | 5.8 | 3 | 2.1 | 0.48 (0.14, 1.62) |
| 6-7 naps/week, <30min | 226 | 13.4 | 90 | 13.3 | 1.04 (0.77, 1.40) | 180 | 13.3 | 26 | 17.8 | **1.77 (1.04, 3.00)** |
| 1-5 naps/week, ≥30 min | 162 | 9.6 | 57 | 8.4 | 1.36 (0.94, 1.96) | 118 | 8.7 | 13 | 8.9 | 1.86 (0.94, 3.70) |
| 6-7 naps/week, ≥30 min | 275 | 16.3 | 140 | 20.7 | 1.28 (0.98, 1.67) | 231 | 17.1 | 40 | 27.4 | **2.42 (1.51, 3.87)** |

^a^OR adjusted for age (continuous), centre (Barcelona, Madrid, Leon, Navarra, Cantabria, Guipuzcoa, Valencia, Huelva, Asturias, Granada, Murcia), and educational level (less than primary, primary, high school, university); ^B^OR additionally adjusted for family history of colorectal cancer or gastric cancer in first degree relatives (yes/no), body mass index (<22.5, 22.5-24.9, 25-29.9, ≥30), leisure time physical activity (inactive, little active, moderately active, very active), smoking status (never, ex-smoker, current smoker) and current occupational status (employed, unemployed, housewife, retired).

*Colorectal cancer: p-for interaction (sleep duration)=0.96, p-for-interaction (daytime napping)=0.46; Gastric cancer: p-for interaction (sleep duration)=0.68, p-for-interaction (daytime napping)=0.09

**Supplemental Table 4.** Sleep duration and daytime napping in relation to colorectal and gastric cancer risk in the MCC-Spain study **by education**.

|  | **Colorectal cancer^a^** | | | | | **Gastric cancer^b^** | | | | |
| --- | --- | --- | --- | --- | --- | --- | --- | --- | --- | --- |
| **Lower education: completed primary education or less** | **Controls**  **(N=1881)** | | **Cases**  **(N=1387)** | | **OR [CI 95%]^c^** | **Controls**  **(N=1675)** | | **Cases**  **(N=311)** | | **OR [CI 95%]^c^** |
| **Sleep duration (hours)** | **(n)** | **%** | **(n)** | **%** |  | **(n)** | **%** | **(n )** | **%** |  |
| ≤5 | 308 | 16.4 | 193 | 13.9 | 0.98 (0.77, 1.26) | 277 | 16.5 | 54 | 17.4 | 1.37 (0.89, 2.12) |
| 6 | 374 | 19.9 | 235 | 16.9 | 1.04 (0.82, 1.31) | 335 | 20.0 | 51 | 16.4 | 1.05 (0.68, 1.63) |
| 7 | 438 | 23.3 | 271 | 19.5 | Ref | 385 | 23.0 | 49 | 15.8 | Ref |
| 8 | 488 | 25.9 | 412 | 29.7 | **1.35 (1.09, 1.66)** | 427 | 25.5 | 89 | 28.6 | **1.55 (1.05, 2.30)** |
| ≥9 | 273 | 14.5 | 276 | 19.9 | **1.42 (1.11, 1.80)** | 251 | 15.0 | 68 | 21.9 | **1.87 (1.22, 2.86)** |
| **Daytime napping frequency and duration combined** |  |  |  |  |  |  |  |  |  |  |
| No naps | 835 | 46.5 | 503 | 39.8 | Ref | 750 | 46.7 | 123 | 40.6 | Ref |
| 1-5 naps/week, <30 min | 83 | 4.6 | 57 | 4.5 | 1.21 (0.83, 1.78) | 63 | 3.9 | 11 | 3.6 | 1.28 (0.64, 2.59) |
| 6-7 naps/week, <30min | 281 | 15.7 | 184 | 14.6 | 1.06 (0.85, 1.34) | 245 | 15.3 | 41 | 13.5 | 1.12 (0.75, 1.69) |
| 1-5 naps/week, ≥30 min | 121 | 6.7 | 91 | 7.2 | 1.24 (0.91, 1.70) | 105 | 6.5 | 24 | 7.9 | 1.36 (0.78, 2.17) |
| 6-7 naps/week, ≥30 min | 476 | 26.5 | 430 | 34.0 | **1.41 (1.17, 1.70)** | 444 | 27.6 | 104 | 34.3 | **1.56 (1.14, 2.15)** |
| **Higher education: completed high school, university or higher** | **Controls**  **(N=1717)** | | **Cases**  **(N=621)** | | **OR [CI 95%]^c^** | **Controls**  **(N=1423)** | | **Cases**  **(N=141)** | | **OR [CI 95%]^c^** |
| **Sleep duration (hours)** | **(n)** | **%** | **(n)** | **%** |  | **(n)** | **%** | **(n)** | **%** |  |
| ≤5 | 164 | 9.6 | 66 | 10.6 | 1.14 (0.81, 1.61) | 142 | 10.0 | 14 | 9.9 | 1.06 (0.55, 2.05) |
| 6 | 349 | 20.4 | 102 | 16.4 | 0.87 (0.65, 1.16) | 290 | 20.4 | 19 | 13.5 | 0.66 (0.37, 1.18) |
| 7 | 610 | 35.5 | 186 | 30.0 | Ref | 490 | 34.4 | 43 | 30.5 | Ref |
| 8 | 499 | 29.1 | 189 | 30.4 | 1.17 (0.91, 1.50) | 424 | 29.8 | 47 | 33.3 | 1.29 (0.82, 1.02) |
| ≥9 | 95 | 5.5 | 78 | 12.6 | **2.31 (1.60, 3.33)** | 77 | 5.4 | 18 | 12.8 | **2.42 (1.27, 4.60)** |
| **Daytime napping frequency and duration combined** |  |  |  |  |  |  |  |  |  |  |
| No naps | 698 | 42.5 | 235 | 40.1 | Ref | 573 | 41.6 | 51 | 37.0 | Ref |
| 1-5 naps/week, <30 min | 110 | 6.7 | 24 | 4.10 | 0.62 (0.38, 1.01) | 84 | 6.1 | 2 | 1.5 | 0.25 (0.06, 1.08) |
| 6-7 naps/week, <30min | 227 | 13.8 | 68 | 11.6 | 0.75 (0.54, 1.05) | 206 | 15.0 | 17 | 12.3 | 0.87 (0.47, 1.60) |
| 1-5 naps/week, ≥30 min | 229 | 13.9 | 75 | 12.8 | 0.90 (0.65, 1.24) | 175 | 12.7 | 20 | 14.5 | 1.21 (0.67, 2.17) |
| 6-7 naps/week, ≥30 min | 377 | 23.0 | 184 | 31.4 | 1.17 (0.91, 1.51) | 339 | 24.6 | 48 | 34.8 | **1.56 (0.98, 2.46)** |

^a^p-for interaction (sleep duration)=0.02, p-for-interaction(daytime napping)=0.07; ^b^p-for interaction(sleep duration)=0.37, p-for-interaction(daytime napping)=0.29

^c^OR adjusted for age (continuous), centre (Barcelona, Madrid, Leon, Navarra, Cantabria, Guipuzcoa, Valencia, Huelva, Asturias, Granada, Murcia),, sex (female, male), and educational level (less than primary, primary, high school, university); ^B^OR additionally adjusted for family history of colorectal cancer or gastric cancer in first degree relatives (yes/no), body mass index (<22.5, 22.5-24.9, 25-29.9, ≥30), leisure time physical activity (inactive, little active, moderately active, very active), smoking status (never, ex-smoker, current smoker) and current occupational status (employed, unemployed, housewife, retired).

**Supplemental Table 5.** Sleep duration and daytime napping in relation to colorectal and gastric cancer risk in the MCC-Spain study **by age at diagnosis (cases) /interview (controls)**

| **Age at diagnosis** | **Colorectal cancer^a^** | | | | | **Gastric cancer^b^** | | | | |
| --- | --- | --- | --- | --- | --- | --- | --- | --- | --- | --- |
|  | **Controls**  **(N=542)** | | **Cases**  **(N=135)** | | **OR [CI 95%]^c^** | **Controls**  **(N=382)** | | **Cases**  **(N=42)** | | **OR [CI 95%]c** |
| ***Age<50 yrs*** | **(n)** | **%** | **(n )** | **%** |  | **(n)** | **%** | **(n )** | **%** |  |
| **Sleep duration (hours)** |  |  |  |  |  |  |  |  |  |  |
| ≤5 | 44 | 8.1 | 11 | 8.1 | 1.45 (0.59, 3.57) | 31 | 7.9 | 5 | 11.9 | 3.19 (0.76, 13.5) |
| 6 | 111 | 20.5 | 29 | 21.5 | 1.18 (0.62, 2.24) | 87 | 22.2 | 11 | 26.2 | 2.22 (0.74, 6.71) |
| 7 | 217 | 40 | 41 | 30.4 | 1.00 (Ref) | 149 | 38 | 9 | 21.4 | 1.00 (Ref) |
| 8 | 146 | 26.9 | 44 | 32.6 | 1.69 (0.95, 3.00) | 109 | 27.8 | 12 | 28.6 | 2.27 (0.73,7.08) |
| ≥9 | 24 | 4.4 | 10 | 7.4 | **2.67 (1.01, 7.07)** | 16 | 4.1 | 5 | 11.9 | **11.02 (2.01, 60.4)** |
| **Daytime napping frequency and duration** |  |  |  |  |  |  |  |  |  |  |
| No naps | 288 | 53.1 | 58 | 43.0 | 1.00 (Ref) | 215 | 54.8 | 13 | 31.0 | 1.00 (Ref) |
| 1-5 naps/week, <30 min | 43 | 8.2 | 12 | 9.5 | 1.18 (0.50, 2.75) | 28 | 7.3 | 4 | 9.8 | 2.63 (0.67, 10.4) |
| 6-7 naps/week, <30min | 32 | 6.1 | 11 | 8.7 | 1.85 (0.74, 4.64) | 26 | 6.8 | 3 | 7.3 | 2.70 (0.62, 14.9) |
| 1-5 naps/week, ≥30 min | 96 | 18.3 | 23 | 18.3 | 1.18 (0.62, 2.23) | 61 | 16 | 13 | 31.7 | 2.64 (0.95, 7.36) |
| 6-7 naps/week, ≥30 min | 65 | 12.4 | 22 | 17.5 | 1.41 (0.70, 2.82) | 52 | 13.6 | 8 | 19.5 | 3.07 (0.91, 10.4) |
| ***Age≥50 yrs*** | **Controls**  **(N=3056)** | | **Cases**  **(N=1873)** | | **OR [CI 95%]^c^** | **Controls**  **(N=2707)** | | **Cases**  **(N=410)** | | **OR [CI 95%]^c^** |
| **Sleep duration (hours)** |  |  |  |  |  |  |  |  |  |  |
| ≤5 | 428 | 14.0 | 248 | 13.2 | 1.02 (0.83, 1.25) | 388 | 14.3 | 63 | 15.4 | 1.22 (0.84, 1.77) |
| 6 | 613 | 20.1 | 308 | 16.4 | 0.94 (0.78, 1.14) | 537 | 19.8 | 59 | 14.4 | 0.85 (0.60, 1.25) |
| 7 | 831 | 27.2 | 416 | 22.2 | 1.00 (Ref) | 727 | 26.9 | 83 | 20.2 | 1.00 (Ref) |
| 8 | 841 | 27.5 | 557 | 29.7 | **1.24 (1.05, 1.47)** | 743 | 27.4 | 124 | 30.2 | **1.40 (1.03, 1.92)** |
| ≥9 | 344 | 11.3 | 344 | 18.4 | **1.56 (1.27, 1.91)** | 312 | 11.5 | 81 | 19.8 | **1.71 (1.19, 2.45)** |
| **Daytime napping frequency and duration** |  |  |  |  |  |  |  |  |  |  |
| No naps | 1245 | 40.8 | 680 | 36.3 | 1.00 (Ref) | 1108 | 42.6 | 161 | 40.3 | 1.00 (Ref) |
| 1-5 naps/week, <30 min | 150 | 5.1 | 69 | 4 | 0.91 (0.67, 1.25) | 119 | 4.6 | 9 | 2.3 | 0.65 (0.32, 1.34) |
| 6-7 naps/week, <30min | 476 | 16.3 | 241 | 14 | 0.95 (0.79, 1.15) | 425 | 16.3 | 55 | 13.8 | 1.00 (0.71, 1.42) |
| 1-5 naps/week, ≥30 min | 254 | 8.7 | 143 | 8.3 | 1.10 (0.87, 1.40) | 219 | 8.4 | 31 | 7.8 | 1.08 (0.70, 1.67) |
| 6-7 naps/week, ≥30 min | 788 | 27 | 592 | 34.3 | **1.36 (1.16, 1.58)** | 733 | 28.2 | 144 | 36.0 | **1.54 (1.16, 1.97)** |

^a^p-for interaction (sleep duration)=0.20, p-for-interaction(daytime napping)=0.29; ^b^p-for interaction(sleep duration)=0.92, p-for-interaction(daytime napping)=0.16

^c^OR adjusted for age, sex, centre (Barcelona, Madrid, Leon, Navarra, Cantabria, Guipuzcoa, Valencia, Huelva, Asturias, Granada, Murcia),, educational level (less than primary, primary, high school, university), family history of colorectal cancer or gastric cancer in first degree relatives (yes/no), body mass index (<22.5, 22.5-24.9, 25-29.9, ≥30), leisure time physical activity (inactive, little active, moderately active, very active), smoking status (never, ex-smoker, current smoker), and current occupational status (employed, unemployed, housewife, retired).

**Supplemental Table 6.** Sleep duration and daytime napping in relation to colorectal cancer risk **according to anatomical site** and **TNM staging** (OR=Odds Ratio, 95%CI=95% confidence interval).

|  | **Anatomical site** | | | | **TNM staging** | | | | | |
| --- | --- | --- | --- | --- | --- | --- | --- | --- | --- | --- |
|  | **Colon cancer^a^** | | **Rectum cancer^a^** | | **Stages O-II^b^** | | **Stage III** | | **Stage IV^b^** | |
|  | **Cases**  **(N=1224)** | **OR**  **(95% CI)^d^** | **Cases**  **(N=761)** | **OR**  **(95% CI)^d^** | Cases  (N=1,027) | OR  (95% CI)^d^ | Cases  (N=542) | OR  (95% CI)^d^ | Cases (N=315) | OR  (95% CI)^d^ |
| **Sleep duration (hours)** |  |  |  |  |  |  |  |  |  |  |
| ≤5 | 168 | 1.13 (0.89, 1.42) | 89 | 0.93 (0.70, 1.24) | 123 | 0.99 (0.77, 1.29) | 80 | 1.11 (0.81, 1.51) | 39 | 1.09 (0.72, 1.65) |
| 6 | 213 | 1.03 (0.84, 1.28) | 121 | 0.88 (0.68, 1.14) | 174 | 1.01 (0.80, 1.27) | 95 | 0.94 (0.70, 1.26) | 46 | 0.88 (0.60, 1.30) |
| 7 | 266 | 1.00 (Ref) | 187 | 1.00 (Ref) | 223 | 1 (Ref) | 132 | 1 (Ref) | 72 |  |
| 8 | 360 | **1.28 (1.06, 1.55)** | 233 | 1.24 (0.99, 1.54) | 330 | 1.38 (1.13, 1.69) | 138 | 1.00 (0.76, 1.29) | 103 | 1.49 (1.08, 2.06) |
| ≥9 | 217 | **1.61 (1.28, 2.03)** | 131 | **1.52 (1.16, 1.99)** | 177 | 1.56 (1.22, 2.00) | 97 | 1.41 (1.03, 1.91) | 55 | 1.83 (1.24, 2.71) |
| **Daytime napping frequency and duration** |  |  |  |  |  |  |  |  |  |  |
| No naps | 455 | 1.00 (Ref) | 273 | 1.00 (Ref) | 376 |  | 210 |  | 105 |  |
| 1-5 naps/week, <30 min | 52 | 0.99 (0.70, 1.39) | 28 | 0.89 (0.58, 1.37) | 44 | 1.00 (0.70, 1.45) | 21 | 0.90 (0.55, 1.46) | 8 | 0.59 (0.28, 1.24) |
| 6-7 naps/week, <30min | 147 | 0.92 (0.73, 1.14) | 102 | 1.05 (0.81, 1.36) | 127 | 0.94 /0.74, 1.88) | 69 | 0.96 (0.71, 1.29) | 36 | 0.96 (0.64, 1.43) |
| 1-5 naps/week, ≥30 min | 100 | 1.11 (0.86, 1.44) | 66 | 1.11 (0.82, 1.51) | 85 | 1.15 (0.87, 1.52) | 41 | 0.97 (0.67, 1.40) | 29 | 1.15 (0.74, 1.79) |
| 6-7 naps/week, ≥30 min | 371 | **1.32 (1.10, 1.57)** | 237 | **1.36 (1.11, 1.68)** | 314 | **1.30 (1.08, 1.52)** | 156 | 1.23 (0.97, 1.56) | 112 | **1.69 (1.25, 2.28)** |

^a^Colon vs Rectum cancer: p-heterogeneity 0.26, 0.29, 0.77, 0.71 for sleep duration categories ( ≤5 hours, 6, 7, 8, ≥9 categories) respectively, p-heterogeneity: 0.68, 0.36, 0.99, 0.74 for daytime napping categories (1-5 naps/week, <30 min, 6-7 naps/week, <30min , 1-5 naps/week, ≥30 min, 6-7 naps/week, ≥30 min) respectively.

^b^Stages 0-II vs Stage IV: p-heterogeneity 0.70, 0.55, 0.68, 0.46 for sleep duration categories (≤5 hours, 6, 7, 8, ≥9 categories) respectively, p-heterogeneity: 0.18, 0.93, 0.99, 0.11 for daytime napping categories (1-5 naps/week, <30 min, 6-7 naps/week, <30min , 1-5 naps/week, ≥30 min, 6-7 naps/week, ≥30 min) respectively.

^c^The numbers of cancer subsites may not be equal to the total number of cancer cases because in some cases the anatomical site or staging was unknown.

^d^OR adjusted for age (continuous), sex, centre (Barcelona, Madrid, Leon, Navarra, Cantabria, Guipuzcoa, Valencia, Huelva, Asturias, Granada, Murcia),, family history of colorectal cancer or gastric cancer in first degree relatives (yes/no), body mass index (<22.5, 22.5-24.9, 25-29.9, ≥30), leisure time physical activity (inactive, little active, moderately active, very active), smoking status (never, ex-smoker, current smoker) , and current occupational status (employed, unemployed, housewife, retired).

**Supplemental Table 7.** Sleep duration and daytime napping in relation to gastric cancer risk **according to anatomical site and Lauren’s classification** (OR=Odds Ratio, 95%CI=95% confidence interval).

|  | **Anatomical site** | | | | **Lauren’s classification** | | | |
| --- | --- | --- | --- | --- | --- | --- | --- | --- |
|  | **Non-cardia^a^** | | **Cardia or Esophageal^a^** | | **Intestinal^b^** | | **Difuse^b^** | |
|  | **Cases**  **(N=1224)** | **OR**  **(95% CI)^d^** | **Cases**  **(N=761)** | **OR**  **(95% CI)^d^** | **Cases**  **(N=176)** | **OR**  **(95% CI)^d^** | **Cases**  **(N=101)** | **OR**  **(95% CI)^d^** |
| Sleep duration (hours) |  |  |  |  |  |  |  |  |
| ≤5 | 53 | 1.44 (0.96, 2.15) | 15 | 1.14 (0.58, 2.21) | 31 | 1.07 (0.59, 1.94) | 22 | 1.43 (0.72, 2.86) |
| 6 | 48 | 0.98 (0.65, 1.47) | 20 | 0.85 (0.47, 1.59) | 22 | 0.96 (0.54, 1.69) | 15 | 0.96 (0.49, 1.86) |
| 7 | 61 | 1.00 (Ref) | 28 | 1.00 (Ref) | 24 | 1.00 (Ref) | 16 | 1.00 (Ref) |
| 8 | 103 | **1.60 (1.14, 2.26)** | 26 | 1.07 (0.61, 1.90) | 52 | 1.43 (0.88, 2.32) | 29 | 1.42 (0.80, 2.53) |
| ≥9 | 62 | **1.94 (1.29, 2.90)** | 22 | **2.27 (1.21, 4.26)** | 38 | **1.83 (1.06, 3.13)** | 19 | **2.44 (1.24, 4.78)** |
| Daytime napping |  |  |  |  |  |  |  |  |
| No naps | 122 | 1.00 (Ref) | 47 | 1.00 (Ref) | 66 | 1.00 (Ref) | 36 | 1.00 (Ref) |
| 1-5 naps/week, <30 min | 8 | 0.78 (0.37, 1.66) | 47 | 0.93 (0.35, 2.47) | 3 | 0.56 (0.17, 1.86) | 5 | 1.45 (0.55, 3.81) |
| 6-7 naps/week, <30min | 44 | 1.13 (0.77, 1.64) | 5 | 0.79 (0.41, 1.52) | 19 | 0.98 (0.56, 1.69) | 14 | 1.24 (0.65, 2.37) |
| 1-5 naps/week, ≥30 min | 32 | 1.54 (0.99, 2.39) | 13 | 0.83 (0.41, 1.70) | 15 | 1.34 (0.71, 2.51) | 11 | 1.49 (0.73, 3.04) |
| 6-7 naps/week, ≥30 min | 115 | **1.75 (1.31, 2.36)** | 11 | 1.01 (0.61, 1.65) | 59 | **1.61 (1.08, 2.41)** | **33** | **1.97 (1.17, 3.31)** |

^a^Non cardia vs Cardia Gastric cancer: p-heterogeneity : 0.54, 0.73, 0.22, 0.67 for sleep duration categories ( ≤5 hours, 6, 7, 8, ≥9), p-heterogeneity: 0.77, 0.33, 0.15, 0.04 for daytime napping categories (1-5 naps/week, <30 min, 6-7 naps/week, <30min , 1-5 naps/week, ≥30 min, 6-7 naps/week, ≥30 min) respectively

^b^Intestinal vs Difuse: p-heterogeneity: 0.52, 0.99, 0.98, 0.49 for sleep duration categories ( ≤5 hours, 6, 7, 8, ≥9), p-heterogeneity: 0.22, 0.57, 0.82, 0.53 for daytime napping categories (1-5 naps/week, <30 min, 6-7 naps/week, <30min , 1-5 naps/week, ≥30 min, 6-7 naps/week, ≥30 min) respectively

^c^The numbers of cancer subsites may not be equal to the total number of cancer cases because in some cases the tumor site and/or classification was unknown.

^d^OR adjusted for age (continuous), sex, centre (Barcelona, Madrid, Leon, Navarra, Cantabria, Guipuzcoa, Valencia, Huelva, Asturias, Granada, Murcia),, family history of colorectal cancer or gastric cancer in first degree relatives (yes/no), body mass index (<22.5, 22.5-24.9, 25-29.9, ≥30), leisure time physical activity (inactive, little active, moderately active, very active), smoking status (never, ex-smoker, current smoker) , and current occupational status (employed, unemployed, housewife, retired).

**Supplemental Table 8.** Sensitivity analysis of sleep duration and daytime napping in relation to colorectal and gastric cancer risk in the MCC-Spain study, **excluding participants with sleep problems in the 5 years prior to recruitment/cancer diagnosis.**

|  | **Colorectal cancer (N=5606)** | | | | | | **Gastric cancer (N=3551)** | | | | | |
| --- | --- | --- | --- | --- | --- | --- | --- | --- | --- | --- | --- | --- |
|  | **Controls**  **(N=2430)** | | **Cases**  **(N=1373)** | | **OR (CI 95%)^a^** | **OR (CI 95%)^b^** | **Controls**  **(N=2134)** | | **Cases**  **(N=331)** | | **OR (CI 95%)^a^** | **OR (CI 95%)^b^** |
| **Sleep duration (hours)** | **(n)** | **%** | **(n )** | **%** |  |  | **(n)** | **%** | **(n )** | **%** |  |  |
| ≤5 | 161 | 6.6 | 86 | 6.3 | 1.00 (0.74, 1.36) | 1.01 (0.74, 1.38) | 149 | 7 | 29 | 8.8 | 1.42 (0.87, 2.30) | 1.46 (0.89, 2.40) |
| 6 | 453 | 18.6 | 188 | 13.7 | 0.86 (0.69, 1.07) | 0.85 (0.68, 1.07) | 405 | 19 | 41 | 12.4 | 0.85 (0.56, 1.28) | 0.79 (0.51, 1.20) |
| 7 | 784 | 32.3 | 341 | 24.8 | 1.00 (Ref) | 1.00 (Ref) | 669 | 31.3 | 71 | 21.5 | 1.00 (Ref) | 1.00 (Ref) |
| 8 | 755 | 31.1 | 472 | 34.4 | **1.29 (1.08, 1.54)** | **1.31 (1.09, 1.58)** | 665 | 31.1 | 120 | 36.3 | **1.54 (1.12, 2.13)** | **1.63 (1.17, 2.28)** |
| ≥9 | 278 | 11.4 | 286 | 20.8 | **1.64 (1.31, 2.05)** | **1.71 (1.35, 2.15)** | 247 | 11.6 | 70 | 21.1 | **1.87 (1.27, 2.75)** | **1.95 (1.31, 2.91)** |
| **Daytime napping frequency (days/week)** | **Controls**  **(N=2366)** | | **Cases**  **(N=1348)** | |  |  | **Controls**  **(N=2096)** | | **Cases**  **(N=326)** | |  |  |
| No naps | 1014 | 42.8 | 473 | 35.1 | 1.00 (Ref) | 1.00 (Ref) | 889 | 42.4 | 122 | 37.4 | 1.00 (Ref) | 1.00 (Ref) |
| 1-2 | 227 | 9.6 | 88 | 6.5 | 0.98 (0.73, 1.30) | 0.94 (0.70, 1.27) | 179 | 8.5 | 27 | 8.3 | 1.46 (0.91, 2.37) | 1.35 (0.82, 2.21) |
| 3-5 | 162 | 6.8 | 107 | 7.9 | **1.33 (1.01, 1.77)** | **1.38 (1.03, 1.84)** | 135 | 6.4 | 20 | 6.1 | 1.12 (0.66, 1.90) | 1.18 (0.69, 2.032) |
| 6-7 | 964 | 40.7 | 680 | 50.4 | **1.32 (1.12, 1.54)** | **1.32 (1.12, 1.56)** | 894 | 42.6 | 157 | 48.2 | **1.30 (0.99, 1.71)** | **1.37 (1.03, 1.81)** |
| *Frequency (cont; per day/week)* |  |  |  |  | *1.04 (1.02, 1.06)* | *1.04 (1.02, 1.07)* |  |  |  |  | *1.03 (1.00, 1.07)* | *1.04 (1.00, 1.09)* |
| **Daytime napping duration (minutes/day)** | **Controls**  **(N=3437)** | | **Cases**  **(N=1851)** | |  |  | **Controls**  **(N=2987)** | | **Cases**  **(N=441)** | |  |  |
| No naps | 1014 | 43.6 | 473 | 37.4 | 1.00 (Ref) | 1.00 (Ref) | 889 | 43.2 | 122 | 37.7 | 1.00 (Ref) | 1.00 (Ref) |
| <15 | 176 | 7.6 | 71 | 5.6 | 0.84 (0.62, 1.15) | 0.89 (0.65, 1.23) | 149 | 7.2 | 21 | 6.5 | 1.08 (0.65, 1.81) | 1.20 (0.71, 2.03) |
| 15-29 | 290 | 12.5 | 151 | 11.9 | 1.08 (0.85, 1.37) | 1.08 (0.85, 1.37) | 255 | 12.4 | 27 | 8.3 | 0.81 (0.51, 1.27) | 0.82 (0.52, 1.31) |
| 30-60 | 384 | 16.5 | 231 | 18.2 | **1.24 (1.01, 1.52)** | **1.25 (1.01, 1.54)** | 352 | 17.1 | 62 | 19.1 | 1.36 (0.96, 1.92) | 1.38 (0.97, 1.98) |
| >60 | 463 | 19.9 | 340 | 26.9 | **1.40 (1.16, 1.53)** | **1.42 (1.17, 1.73)** | 413 | 20.1 | 92 | 28.4 | **1.75 (1.28, 2.40)** | **1.86 (1.34, 2.57)** |
| *Duration (cont; per 30minutes napping)* |  |  |  |  | *1.14 (1.07, 1.22)* | *1.15 (1.07, 1.22)* |  |  |  |  | *1.19 (1.08, 1.32)* | *1.19 (1.08, 1.32)* |
| **Daytime napping frequency and duration combined** |  |  |  |  |  |  |  |  |  |  |  |  |
| No naps | 1014 | 43.6 | 473 | 37.4 | 1.00 (Ref) | 1.00 (Ref) | 889 | 43.2 | 122 | 37.7 | 1.00 (Ref) | 1.00 (Ref) |
| 1-5 naps/week, <30 min | 176 | 7.6 | 71 | 5.6 | 0.99 (0.70, 1.40) | 1.02 (0.72, 1.47) | 149 | 7.2 | 21 | 6.5 | 1.00 (0.51, 1.95) | 1.06 (0.53, 2.09) |
| 6-7 naps/week, <30min | 290 | 12.5 | 151 | 11.9 | 0.99 (0.79, 1.25) | 1.01 (0.80, 1.28) | 255 | 12.4 | 27 | 8.3 | 0.89 (0.59, 1.33) | 0.93 (0.61, 1.41) |
| 1-5 naps/week, ≥30 min | 384 | 16.5 | 231 | 18.2 | 1.19 (0.92, 1.53) | 1.18 (0.90, 1.53) | 352 | 17.1 | 62 | 19.1 | 1.49 (0.97, 2.29) | 1.45 (0.93, 2.24) |
| 6-7 naps/week, ≥30 min | 463 | 19.9 | 340 | 26.9 | **1.39 (1.16, 1.66)** | **1.41 (1.17, 1.69)** | 413 | 20.1 | 92 | 28.4 | **1.59 (1.18, 2.14)** | **1.70 (1.25, 2.31)** |

^a^OR adjusted for age (continuous), centre (Barcelona, Madrid, Leon, Navarra, Cantabria, Guipuzcoa, Valencia, Huelva, Asturias, Granada, Murcia), sex (female, male), and educational level (less than primary, primary, high school, university);

^b^OR additionally adjusted for family history of colorectal cancer or gastric cancer in first degree relatives (yes/no), body mass index (<22.5, 22.5-24.9, 25-29.9, ≥30), leisure time physical activity (inactive, little active, moderately active, very active), smoking status (never, ex-smoker, current smoker) and current occupational status (employed, unemployed, housewife, retired).
